# Single‐nucleus RNA sequencing reveals ferroptosis as a potential contributor to the pathogenesis of focal cortical dysplasia

**DOI:** 10.1002/ctm2.70668

**Published:** 2026-04-29

**Authors:** Qingyang Zeng, Fengjun Zhu, Dezhi Cao, Yang Sun, Lin Li, Zeshi Tan, Cong Li, Xiaofan Ren, Yidi Liu, Zhiqiang Lin, Dongfang Zou

**Affiliations:** ^1^ Epilepsy Center and Department of Neurology Shenzhen Children’s Hospital Shenzhen Guangdong China; ^2^ Shenzhen Pediatrics Institute Shantou University Medical College Shenzhen Guangdong China

**Keywords:** single‐nucleus RNA sequencing, focal cortical dysplasia, ferroptosis, inhibitory neuron, excitatory neurons, astrocytes, ferritin light chain

## Abstract

**Objective:**

Focal cortical dysplasia (FCD) is a leading cause of drug‐resistant epilepsy, whereas its molecular and cellular mechanisms remain poorly understood. This study aimed to characterize the cellular heterogeneity of FCD and investigate the function of ferroptosis in FCD pathogenesis.

**Methods:**

Single‐nucleus RNA sequencing was carried out on epileptogenic cortical tissues from 18 patients with FCD and 6 perilesional control samples with normal histology. Data were analysed using uniform manifold approximation and projection for dimensionality reduction and visualization. Differentially expressed genes (DEGs) were identified and subjected to Gene Ontology and Kyoto Encyclopedia of Genes and Genomes pathway enrichment analyses. UCell scoring and gene set enrichment analysis (GSEA) were applied to assess pathway activity. Expression levels of ferroptosis‐related genes (FRGs) were validated by immunofluorescence, and biochemical assays quantified the levels of superoxide dismutase (SOD), glutathione (GSH), malondialdehyde (MDA) and lipid peroxides (LPO).

**Results:**

A total of 170 747 nuclei were profiled, resolving five major cell types, including inhibitory neurons, excitatory neurons, astrocytes, microglia and oligodendrocytes. DEGs across these populations were significantly enriched in ferroptosis and oxidative stress–associated pathways. UCell and GSEA highlighted remarkable alterations in ferroptosis, apoptosis and oxidative stress, particularly in inhibitory neurons and astrocytes. Immunofluorescence confirmed upregulation of key FRGs, including ferritin light chain, ferritin heavy chain 1, poly rC binding protein 1, microtubule‐associated protein 1 light chain 3B and prion protein‐encoding gene, in FCD tissues. Concordantly, biochemical assays demonstrated reduced SOD and GSH levels, alongside elevated MDA and LPO levels, confirming the transcriptional and histological findings.

**Conclusions:**

The results indicated that ferroptosis may play a notable role or act as a concurrent mechanism in the pathogenesis of FCD, potentially contributing to the neuronal and glial dysfunction and epileptogenesis. Integrating transcriptomic, histological and biochemical data, this study demonstrated that targeting ferroptosis‐related pathways may hold promise as a potential therapeutic strategy for FCD, providing new insights into the molecular mechanisms underlying this condition.

**Highlights:**

- This study pioneers the first single‐nucleus transcriptomic atlas for Focal Cortical Dysplasia (FCD) types I and II, deciphering the cellular heterogeneity across five major brain cell types within the epileptogenic cortex.
- Through integrated multi‐omics analysis, it reveals for the first time a significant association between the ferroptosis pathway and FCD pathogenesis.
- We identify and validate ferroptosis‐related genes (e.g., *FTH1*, *FTL*, *PCBP1*) as potential biomarkers and therapeutic targets, supported by congruent biochemical evidence of oxidative stress in this drug‐resistant epilepsy.

## Graphical abstract

1. A single‐cell analysis was conducted to investigate the transcriptomic changes in cells within focal cortical dysplasia (FCD).

2. Ferroptosis may play a significant role in the pathogenesis of FCD, potentially contributing to cellular damage and epileptogenesis.

3. Ferroptosis‐related biomarkers (*FTL*, *FTH1* and *PCBP1*) were validated, demonstrating that targeting ferroptosis‐related pathways may hold promise as a therapeutic strategy for FCD.

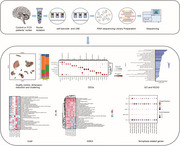

## 1 BACKGROUND

Focal cortical dysplasia (FCD) represents a leading cause of drug‐resistant epilepsy, which is characterized by cortical malformation and abnormal cellular architecture, leading to recurrent seizures and substantial morbidity.^1^ The socio‐economic burden of FCD is noticeable, arising not only from the direct costs of medical care and surgical interventions but also from its remarkable impact on patients’ quality of life and productivity. Despite extensive research into the structural and functional alterations associated with FCD, the cell‐type–specific molecular mechanisms underlying its pathogenesis remain poorly understood, representing a critical gap in the current knowledge.

Although surgical resection achieved seizure freedom in approximately 50%–70% of patients,^2^ a remarkable proportion of patients remain refractory to treatment, underscoring the urgent need to elucidate the molecular mechanisms underlying FCD pathogenesis. Although previous studies have identified oxidative stress and apoptosis as key contributors,^3^, ^4^ the specific function of ferroptosis, a regulated iron‐dependent cell death pathway characterized by lipid peroxidation, in FCD has not yet been investigated. Ferroptosis is a programmed cell death driven by disruptions in iron metabolism, lipid metabolism and redox homeostasis.^5^, ^6^ Emerging evidence indicates that ferroptosis could regulate neuronal cell death in diverse neurological disorders, including epilepsy, and the pharmacological inhibition of ferroptosis exerts neuroprotective effects against damage resulting from seizures.^7^, ^8^ The potential roles and underlying mechanisms of ferroptosis in FCD were investigated.

Single‐nucleus RNA sequencing (snRNA‐seq) presents single‐cell resolution and enables the comprehensive dissection of cellular heterogeneity and transcriptional dynamics in epileptogenic brain tissues. This powerful approach is particularly valuable for elucidating the unresolved molecular pathologies of FCD. Notably, although germline and somatic mutations in the mTOR pathway account for 59% of FCD type II cases,^9^, ^10^ the molecular aetiologies of FCD I and residual FCD II remain elusive. Recent snRNA‐seq research on FCD IIIa highlighted dysregulated excitatory neurons and oligodendrocyte precursor cells;^11^ however, comparable cell‐type‐resolved investigations of FCD types I and II are currently lacking.

By profiling 18 FCD lesions and 6 perilesional controls, this study aimed to (1) delineate the transcriptomic landscape of FCD across both neuronal and non‐neuronal subclusters and (2) investigate the potential contribution of ferroptosis to FCD pathogenesis through integrated differential expression, Gene Ontology (GO), Kyoto Encyclopedia of Genes and Genomes (KEGG), gene set enrichment analysis (GSEA) and UCell scoring. Consequently, these analyses provide insights into the molecular mechanisms of FCD and demonstrate that ferroptosis may represent a previously underappreciated mechanism contributing to disease progression. The results also indicated that targeting ferroptosis‐related pathways could provide a potential direction for managing refractory epilepsy, although further investigation is needed to fully elucidate its therapeutic potential.

## 2 MATERIALS AND METHODS

### 2.1 Human sample collection

Between June and December 2023, a total of 18 patients diagnosed with FCD were recruited from the surgery division of the epileptic centre affiliated to Shenzhen Children's Hospital. This study was approved by the Medical Ethics Committee of the Shenzhen Children's Hospital (Approval No. 202308102), and informed consent was obtained from participants’ parents or their legal guardians.

Prior to surgical resection of the epileptogenic focus, each participant underwent standardized preoperative assessments, including a detailed review of medical records, neurological and neuropsychological examinations, high‐resolution magnetic resonance imaging (MRI) (3.0T), video electroencephalography (EEG) and F18‐fluorodeoxyglucose positron emission tomography‐computed tomography (F18 FDG‐PET/CT).

The inclusion criteria were summarized as follows: (1) patients’ age between 0 and 18 years; (2) diagnosis of drug‐resistant epilepsy according to standard criteria; (3) MRI evidence suggestive of FCD; (4) EEG evidence of focal epileptic discharges and seizures originating from a localized region corresponding to MRI findings; and (5) PET‐CT findings demonstrating focal hypometabolism closely aligning with the MRI lesion. The exclusion criteria were summarized as follows: neuropsychological evidence of intellectual developmental delay that could confound the diagnosis of FCD. All recruited children were of Chinese ethnicity.

The highly epileptogenic cortical tissues were precisely localized using preoperative stereoelectroencephalography (SEEG) and further reconfirmed by intraoperative electrocorticogram (ECoG) recording.^12^, ^13^ Highly epileptogenic activity was defined by the presence of consistent spike morphology and distribution, with a frequency exceeding 1 spike per 10 s in ECoG recordings. Control tissues were characterized by the absence of epileptic discharges on SEEG and ECoG, as well as by the lack of significant histopathological abnormalities. Applying these selection criteria, highly epileptogenic cortical tissues were obtained from 18 patients with FCD, including 7 with FCD type Ia, 3 with FCD type Ic, 4 with FCD type IIa and 4 with FCD type IIb. Additionally, six epileptogenic perilesional tissues with normal pathology were used as controls. All diagnoses were ultimately confirmed by postoperative neuropathological examination. The neuropathological diagnosis of FCD was made in accordance with the criteria set forth by the International League Against Epilepsy (ILAE) criteria.^14^ Comprehensive neuropathological assessments were conducted, involving evaluations via haematoxylin and eosin staining, as well as immunohistochemical analyses utilizing markers, such as neuronal nuclei, glial fibrillary acidic protein (GFAP), neurofilament, vimentin, nestin and oligodendrocyte transcription factor 2 (OLIG2).

### 2.2 Nuclei isolation and sorting from brain tissue

Brain tissue samples were carefully harvested within 10 min of resection to preserve freshness and were immediately frozen in liquid nitrogen for subsequent processing. Before further processing, FCD cortical fragments were washed in ice‐cold PBSE (phosphate‐buffered saline containing 2 mM EGTA) to remove blood contamination and tissue debris. Mechanical dissociation was performed, followed by treatment with the GEXSCOPE Nucleus Separation reagent (Singleron Biotechnologies), in accordance with the manufacturer's instructions. The isolated nuclear suspension was diluted to a concentration of 2.5 × 10^6^ nuclei per millilitre in PBSE, which was filtered through a 40 µm cell strainer, and assessed for viability using trypan blue staining. For fluorescence‐activated sorting, nuclei were stained with 1 µg/mL DAPI (4′,6‐diamidino‐2‐phenylindole, D1306; Thermo Fisher Scientific), which enabled the selection of intact, single DAPI‐positive nuclei. Potential doublets were identified based on the expression levels of established cellular markers. Clusters co‐expressing multiple lineage‐specific markers were excluded from downstream analysis to ensure data accuracy and interpretability.

To minimize the effects of RNA contamination and cell doublets on downstream analyses, a two‐pronged approach was employed. First, DecontX was utilized to accurately estimate and effectively remove any contamination. Subsequently, DoubletFinder was deployed to identify and eliminate cell doublets, thereby enhancing the precision and robustness of the results.

Moreover, cDNA samples were considered qualified for subsequent library preparation only if they met the following criteria: (1) a total quantity >30 ng as measured by Qubit quantification (or >260 ng for full‐length immuno‐panels); (2) a prominent peak between 900 and 2000 bp on the bioanalyser profile; and (3) a fragment proportion between 1000 and 5000 bp greater than 15%. All libraries were constructed using samples that met these stringent quality control standards.

### 2.3 Single‐nucleus RNA‐seq library construction

The nuclear suspension was adjusted to a concentration of 3–4 × 10^5^ nuclei per millilitre in ice‐cold PBS and promptly loaded into the microfluidic cartridge of the GEXSCOPE Single‐Nucleus RNA‐seq platform (Singleron Biotechnologies) for nuclear barcoding and cDNA library preparation. Notably, the snRNA‐seq libraries were constructed in strict accordance with the manufacturer's instructions. The resulting libraries were thereafter sequenced on an Illumina NovaSeq 6000 platform, yielding 150 bp paired‐end reads.

### 2.4 Primary analysis of raw read data (snRNA‐seq)

Raw sequencing reads were processed using CeleScope software (version 1.5.2, Singleron) to generate gene expression count matrices. Cell barcodes and unique molecular identifiers (UMIs) were extracted and corrected from Read 1 (R1). Adapter and poly(A) sequences were trimmed from Read 2 (R2), followed by alignment to the GRCh38 human reference genome with STAR (ver. 2.6.1b). Uniquely mapped reads were assigned to genes using FeatureCounts (ver. 2.0.1) with the −*t* gene parameter to include both exonic and intronic reads. Reads with identical barcodes, UMIs and gene annotations were collapsed to generate the final digital gene expression matrix. Multimapping or ambiguous reads were discarded and excluded from downstream analysis. Sequencing produced an average depth of approximately 403 million reads per sample. The median number of detected genes per nucleus ranged from 681 to 2320 across samples, with UMI counts consistently around twice the corresponding gene counts, consistent with expectations based on the applied sequencing depth. This depth has been validated in previous studies as being sufficient for reliable transcriptional profiling. The analysis incorporated pre‐RNA annotation by aligning sequencing reads to a reference genome, enabling the identification of intronic regions (intronic reads) to distinguish between non‐spliced pre‐RNA and spliced mature RNA.

### 2.5 Quality control, dimension reduction and clustering (Scanpy)

All downstream analyses were performed using Scanpy 1.8.1 in Python 3.7. Each sample was filtered according to the following criteria: cells with ≥200 detected genes while excluding the top 2% by gene count; the top 2% of cells by UMI count were also removed; cells with >5% mitochondrial reads were excluded; and genes detected in ≥5 cells were retained. After these steps, 170 747 high‐quality cells remained, with a median of 1743 genes and 4191 UMIs per cell. Counts were library‐size normalized, log‐transformed and the 2000 most variable genes were identified (Seurat flavour). Principal component analysis (PCA) of the scaled variable‐gene matrix yielded the first 20 principal components for graph construction. Louvain clustering (resolution = 1.2) resolved 26 distinct groups, which were visualized by uniform manifold approximation and projection (UMAP).^15^ The resolution value of 1.2 was specifically utilized to optimize clustering resolution during dimensionality reduction. This parameter exclusively dictates the number of subgroups generated in the dimensionality reduction process, without any impact on the total cellular content or gene expression levels in the dataset. Given the large sample size and substantial cellular quantity in this project, this parameter configuration promoted the identification of more distinct and biologically meaningful subgroups, thereby facilitating more detailed and refined data annotation and downstream analyses.

To ensure that the sequencing depth was sufficient for reliable gene detection across all cell types, including rare subpopulations, read distribution and data saturation were assessed. The mean number of reads per cell across samples ranged from 18 100 to 291 237, falling within the expected range for droplet‐based snRNA‐seq. Crucially, sequencing saturation analysis revealed a clear plateau in novel gene discovery with increasing depth, confirming data saturation (Figure S1). Additionally, the alignment strategy precisely distinguished non‐spliced pre‐mRNAs from mature RNAs, ensuring accurate nuclear transcriptome quantification. Collectively, these analyses indicated that the sequencing depth was adequate to mitigate the risk of technical under‐sampling for biologically relevant signals.

### 2.6 RNA velocity

To infer transcriptional dynamics, BAM files that contained neuronal and glial transcriptomes were processed jointly with the GRCh38 reference genome using velocyto (ver. 0.23) and scVelo (ver. 0.17.17) under default settings in Python. The resulting RNA velocity vectors were projected onto the previously generated Seurat UMAP embedding to ensure coherent visualization.^16^

### 2.7 Batch‐effect correction

Potential inter‐sample technical variation was mitigated using Harmony (ver. 1.0) (theta = 2, sigma = .1, lambda = 1), which integrated the first 20 principal components from PCA to generate a harmonized embedding free of batch‐specific artefacts.^17^

### 2.8 Subtyping of major cell types

To generate a high‐resolution transcriptional map of excitatory and inhibitory neurons, cells from the corresponding clusters were extracted and subjected to re‐clustering for subtype‐level analysis. The above‐described preprocessing and dimensionality reduction steps were applied, and the clustering resolution was set at 1.2. Similarly, to resolve subtypes of astrocytes, microglial cells and oligodendrocytes, the clustering resolution was adjusted to .5.

### 2.9 Cell‐type annotation

Clusters were annotated by comparing cluster‐specific marker genes with the SynEcoSys knowledgebase (Singleron), a curated resource integrating canonical signatures from CellMarkerDB, PanglaoDB and recent snRNA‐seq studies.

### 2.10 Analysis of differentially expressed genes (DEGs) using Scanpy

DEGs, serving as cluster‐ and subcluster‐specific markers, were identified using a robust multi‐step computational framework implemented with the scanpy.tl.rank_genes_groups function. Differential expression analysis was performed at the single‐cell level by comparing each subcluster against all remaining cells using the Wilcoxon rank‐sum test, a non‐parametric approach well suited for sparse single‐cell expression data. Identified genes were then subjected to stringent statistical filtering (Benjamini–Hochberg adjusted FDR < .05), ranked by average log_2_ fold‐change, and further restricted to genes expressed in >10% of cells in at least one comparison group with a mean absolute log_2_ fold‐change >1. Finally, top candidate markers were cross‐validated against established cell marker databases and the published literature, ensuring biological plausibility beyond statistical significance. Collectively, this strategy ensured that cluster and subcluster annotations were grounded in statistically robust and biologically informed marker gene expression.

### 2.11 Pathway enrichment analysis

The biological roles of neuronal and glial DEGs were interrogated using clusterProfiler (ver. 3.16.1) package in R. GO terms covering biological process (BP), cellular component (CC) and molecular function (MF), along with KEGG pathways, were evaluated, in which enrichments were considered significant at FDR < .05.^17^ GSEA was undertaken using the clusterProfiler R package against the molecular signatures database (MSigDB) hallmark gene sets.^18^ Neuronal and glial DEG lists were ranked by |log_2_FC|, and pathways with *p* < .05 were considered significantly enriched.

### 2.12 UCell gene set scoring

Enrichment scores for neuronal‐ and glial‐derived DEG signatures were calculated using UCell (ver. 1.1.0). The package applied Mann–Whitney *U* test to rank query genes in each cell, and a per‐cell signature score was thereafter calculated.^19^

### 2.13 Transcription factor (TF) regulatory network analysis using pySCENIC

Transcription factor regulatory networks were reconstructed using pySCENIC 0.11.0, with the snRNA‐seq expression matrix and the AnimalTFDB TF list as inputs. Regulon discovery was carried out in three steps. First, GRNBoost2 was applied to infer co‐expression links between TFs and their putative target genes. Second, cisTarget refined the network by pruning indirect edges based on motif enrichment, producing high‐confidence regulons. Finally, AUCell quantified regulon activity in each cell, and regulons were ranked within clusters via regulon specificity scores (RSS) and visualized as heat maps.^20^

### 2.14 Dual‐label immunofluorescence

Cryopreserved tissue sections (15 specimens from highly epileptogenic FCD cortices and 5 specimens from adjacent histologically normal cortices) were cut at 8 µm thickness and air‐dried for 20–30 min. Sections were fixed in 4% paraformaldehyde for 30 min, followed by thrice washing with PBS (5 min each). Antigen retrieval was performed in EDTA (pH 8.0) using a microwave: high power for 3 min, followed by a 10 min cool‐down and low power for 3 min. Sections were blocked with 5% BSA for 30 min, incubated with primary antibody overnight at 4°C, washed with PBST, and then incubated with Alexa‐conjugated secondary antibody for 30 min at 37°C. Following another washing, the second primary antibody was applied overnight at 4°C and visualized using its respective fluorescent secondary antibody under identical conditions. Nuclei were labelled with DAPI for 10 min, and the sections were mounted with antifade medium. Imaging was carried out using either a fluorescence microscope or a digital slide scanner.

### 2.15 Detection of the levels of SOD, GSH, MDA, LPO

To assess key ferroptosis‐related proteins in FCD, biochemical assays were performed to detect levels of superoxide dismutase (SOD), glutathione (GSH), malondialdehyde (MDA) and lipid hydroperoxides (LPO). Commercial kits (Solarbio: BC5165, BC1175, BC0025, BC5245) were utilized according to the manufacturers’ protocols. Briefly, cells exposed to air or nitrogen plasma for 0–10 min were mixed with ice‐cold extraction buffer at the recommended ratio, lysed and incubated with the corresponding chromogenic reagents. Optical densities were measured at 450, 412, 532 and 600 nm to determine SOD activity and the concentrations of GSH, MDA and LPO.^21^, ^22^, ^23^, ^24^

### 2.16 Statistical analysis

Group comparisons for continuous variables were performed using either the independent *t*‐test or the Wilcoxon rank‐sum test, depending on data distribution and variance assumptions. All statistical computations and graphical illustrations were carried out using R software. The specific tests applied in each analysis are detailed in the respective figure legends. Statistical significance was defined as a two‐tailed *p*‐value of less than .05.

## 3 RESULTS

### 3.1 Patients’ demographic and clinical characteristics

A total of 18 patients with FCD were enrolled, comprising 7 with type Ia, 3 with type Ic, 4 with type IIa and 4 with type IIb. The cohort included 13 men and 5 women. Age at seizure onset ranged from .3 to 13.5 (mean, 3.35) years. All patients presented with focal seizures, with frequencies ranging from 1 to 2 episodes per month to 20–30 per day. Among them, three patients also experienced secondary generalized tonic–clonic seizures. Seizure types were classified in strict accordance with standardized ILAE criteria.^14^ Surgical resection was performed at a median age of 8.45 (range, 3.3–16.4) years, involving the frontal (*n* = 9), temporal (*n* = 6), parietal (*n* = 6), occipital (*n* = 6) and insular (*n* = 3) lobes. Notably, 10 patients underwent multilobar procedures. The core tissue from the primary epileptogenic region in a single lobe was specifically selected and analysed, rather than sampling tissue from all affected lobes. After surgery, the majority of patients (14 in total) achieved Engel Class I A status. Additionally, one patient achieved Class I B, one achieved Class I D, and two achieved Class II B. Postoperative outcomes were classified according to Engel's Classification of Postoperative Outcome.^25^ Moreover, one patient was identified as carrying a pathogenic mutation in the *DEPDC5* gene. Genetic testing was either not performed in the remaining individuals or yielded negative results (Table 1).

**TABLE 1:** Clinical and neuropathological characteristics of patients with focal cortical dysplasia (FCD) after surgery.

ID and neuropathologic diagnosis	Sex	Age at seizure onset (*y*)	Seizure type and seizure frequency	Duration of epilepsy (*y*)	Antiseizure medications	Age at surgery (*y*)	Site of surgery	PO	Gene mutation
1. FCD la	M	.9	FS 6–7 times/day	3.8	OXC, LEV, VPA, LTG	4.9	F, T	ll B	Negative
2. FCD la	M	8	FS 8 times/day, sGTCS once	2.8	OXC, LEV, LCM, PER	11.5	F	l A	NA
3. FCD la	M	13.5	FS 1–3 times/month	2.5	OXC, LEV, PER, VPA, LTG, CZP	16.1	T, P, O	l A	NA
4. FCD la	F	.9	FS 6–8 times/month	14	LTG, LEV, LCM, NZP, PER	13.8	T, P, O	l D	Negative
5. FCD la	M	4.7	FS 20–30 times/day	.4	LEV, LCM, PB, VPA, CZP, OXC, PER	5.1	I	l A	NA
6. FCD la	M	4.1	FS 20 times/day	3.4	OXC, LEV, VPA, TPM, CZP, PER, LCM	7.5	F	l A	NA
7. FCD la	M	5.5	FS 10 times/day	10.9	CBZ, LTG, NZP, OXC, VPA, ZNS	16.4	F	l A	NA
8. FCD lc	M	.3	FS 3–4 times/day	9.6	VPA, LEV, OXC, NZP	10.1	T, P, O	l A	*DEPDC5*
9. FCD lc	M	7.8	FS 12–16 times/month, sGTCS 3–4 times/month	3.5	VPA, OXC, LEV, PER, ZNS, LCM	10.5	P	l B	NA
10. FCD lc	M	3.7	FS 10–30 times/day	3.2	OXC, VPA, TPM, PER, LEV	6.9	F, I	l A	NA
11. FCD lla	M	2	FS 8–9 times/day	8.5	OXC, LCM, LEV, PER	10.5	F	l A	NA
12. FCD lla	F	.3	FS 4–5 times/day	11.3	OXC, TPM, LTG, VPA, PER, LCM, LEV	11.6	F, T, O	l A	NA
13. FCD lla	M	4.4	FS 1–2 times/day	4.1	LCM, OXC, LEV	8.5	F, I	l A	NA
14. FCD lla	F	.9	FS 2 times/month, sGTCS 1 times/year	7.2	LEV, LTG	8.1	O	ll B	NA
15. FCD llb	M	4.9	FS 1–4 times/month	3.5	LEV, VPA, LCM	8.4	T, P, O	l A	Negative
16. FCD llb	F	3	FS 1–2 times/day	3	OXC, LEV, LCM, PER	6.6	F	l A	Negative
17. FCD llb	F	.5	FS 4–6 times/month	3	VPA, OXC, LCM, TPM	3.5	F	l A	NA
18. FCD llb	M	1.2	FS 5–6 times/day	2.1	VPA, LEV	3.3	F	l A	NA

Abbreviations: CBZ, carbamazepine; CLB, clobazam; CZP, clonazepam; F, female; F, frontal lobe; FS, focal seizure; I, insular lobe; IA, completely seizure‐free since surgery; IB, nondisabling simple partial seizures only since surgery; ID, generalized convulsions with AED discontinuation only; ID, identification number; IIB, rare disabling seizures since surgery; LCM, lacosamide; LEV, levetiracetam; LTG, lamotrigine; M, male; mo, month; NA, not available; NZP, nitrazepam; O, occipital lobe; OXC, oxcarbazepine; P, parietal lobe; PB, phenobarbital; PER, perampanel; PO follow‐up 2 years after surgery according to Engel's classification; PO, postoperative outcome (Engel's classification); sGTCS, secondary generalized tonic‐clonic seizure; T, temporal lobe; TPM, topiramate; VPA, valproate; y/yr, years; ZNS, zonisamide.

Statistical comparisons of baseline characteristics across FCD subtypes are summarized in Table 2. Gender distribution was compared using the Fisher's exact test. Ages at the time of seizure onset and surgery, as non‐normally distributed variables, were compared using the Kruskal–Wallis H test. The analyses revealed no significant differences in gender (*p* = .322), age at the time of seizure onset (*p* = .403) or age at the time of surgery (*p* = .160) among the subtypes, confirming that the groups were well‐balanced at baseline (Table 2).

**TABLE 2:** Baseline characteristics of patients with different focal cortical dysplasia (FCD) subtypes.

Characteristic	FCD la (*n* = 7)	FCD lc (*n* = 3)	FCD lla (*n* = 4)	FCD llb (*n* = 4)	*p*‐value
Gender, *n* (%)					
Male	6 (85.7)	3 (100)	2 (50.0)	2 (50.0)	.322
Female	1 (14.3)	0 (0)	2 (50.0)	2 (50.0)	
Age at the time of seizure onset (years)					
Median	4.7	3.7	1.45	2.1	.403
Range	(.9–13.5)	(.3–7.8)	(.3–4.4)	(.5–4.9)	
Age at the time of surgery (years)					
Median	11.5	10.1	9.5	5.05	.160
Range	(4.9–16.4)	(6.9–10.5)	(8.1–11.6)	(3.5–8.4)	

### 3.2 A high‐resolution cellular landscape of FCD

**FIGURE 1:**
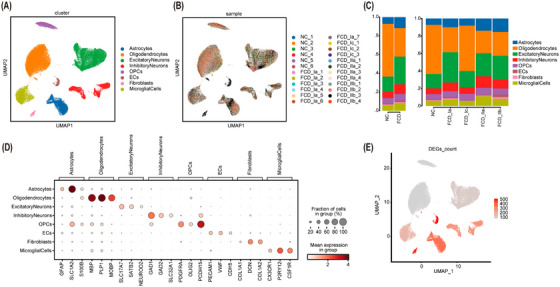
Single nucleus RNA‐sequencing of human brain tissue recapitulated cell‐type‐specific marker genes and cell‐type‐specific changes in focal cortical dysplasia (FCD). (A and B) Uniform manifold approximation and projection (UMAP) projections of 170 747 nuclei from FCD lesions and perilesional controls. Nuclei were coloured by annotated cell type and individual sample origin. (C) Proportion of cells belonging to each cell type in different diagnoses. Proportion of cells belonging to each cell type in each diagnostic category. (D) Integration of top 3 marker gene dot plots for each cell type. (E) UMAP was coloured by the number of differentially expressed genes (DEGs) identified between disease and control groups. ECs, endothelial cells; GFAP, glial fibrillary acidic protein; OPCs, oligodendrocyte progenitor cells.

Highly epileptogenic cortical tissues were collected from 18 patients with FCD, along with perilesional samples showing normal histology as controls. After stringent quality control, 170 747 single‐nucleus transcriptomes were retained for downstream analysis. UMAP revealed 8 transcriptionally distinct cell types, including inhibitory neurons, excitatory neurons, astrocytes, microglia, oligodendrocytes, oligodendrocyte progenitor cells (OPCs), endothelial cells (ECs) and fibroblasts (Figure 1A). Comparative analyses across FCD subtypes (subtypes Ia, Ic, IIa and IIb) and controls revealed broadly consistent subclustering, indicating robust integration of datasets (Figure S2A–F). Cell‐type identities were assigned using canonical marker gene sets. To track tissue origin, nuclei from individual samples were colour‐coded in the UMAP embedding (Figure 1B).

Relative cell type distributions were subsequently compared between FCD patients, including subtypes, and controls. Despite the lack of statistical significance, the FCD group exhibited a notable trend of increased proportion of astrocytes, inhibitory and excitatory neurons, as well as a decrease in oligodendrocytes, compared with the control group. Notably, these cell types displayed a consistent distribution pattern across various disease subtypes (Figure 1C).

Marker genes were identified for each cell type: *GAD1*, *GAD2* and *SLC32A1* for inhibitory neurons; *SLC17A7*, *SATB2* and *NEUROD2* for excitatory neurons; *GFAP*, *SLC1A2* and *S100B* for astrocytes; *CX3CR1*, *P2RY12* and *CSF1R* for microglia; *MBP*, *PLP1* and *MOBP* for oligodendrocytes; *PCDH15*, *PDGFRA* and *OLIG2* for OPCs; *PECAM1*, *VWF* and *CDH5* for ECs; and *COL1A1*, *DCN* and *COL1A2* for fibroblasts (Figure 1D). These marker genes were selected based on their well‐documented roles in defining the specific cell types and their consistent expression patterns in various studies. For instance, *GAD1* and *GAD2* are canonical markers for inhibitory neurons, whereas *SLC17A7* and *SATB2* are widely recognized for excitatory neurons. Similarly, *GFAP* and *S100B* are well‐established markers for astrocytes, and *CX3CR1* and *P2RY12* are specific markers for microglia. Furthermore, these markers have been shown to be conserved across different developmental stages, including paediatric cortex tissue. Studies have demonstrated their reliable expression levels in paediatric samples, ensuring their validity for usage in the present study. This selection ensures that the findings are comparable to existing literature and relevant to the paediatric context.

Taken together, the analysis delineated eight transcriptionally distinct clusters, capturing both cell‐type‐specific and shared gene expression patterns in FCD and control brain tissues. The cell types exhibiting the most DEGs between the control and FCD groups included astrocytes, inhibitory neurons, fibroblasts and ECs (Figure 1E).

### 3.3 The biological roles of the DEGs

To elucidate the biological roles of the DEGs, GO and KEGG pathway enrichment analyses were performed on the most significantly dysregulated genes, which were predicted to contribute to disease‐associated cellular processes. In parallel, GSEA was employed to identify additional enriched pathways relevant to the FCD pathogenesis.

**FIGURE 2:**
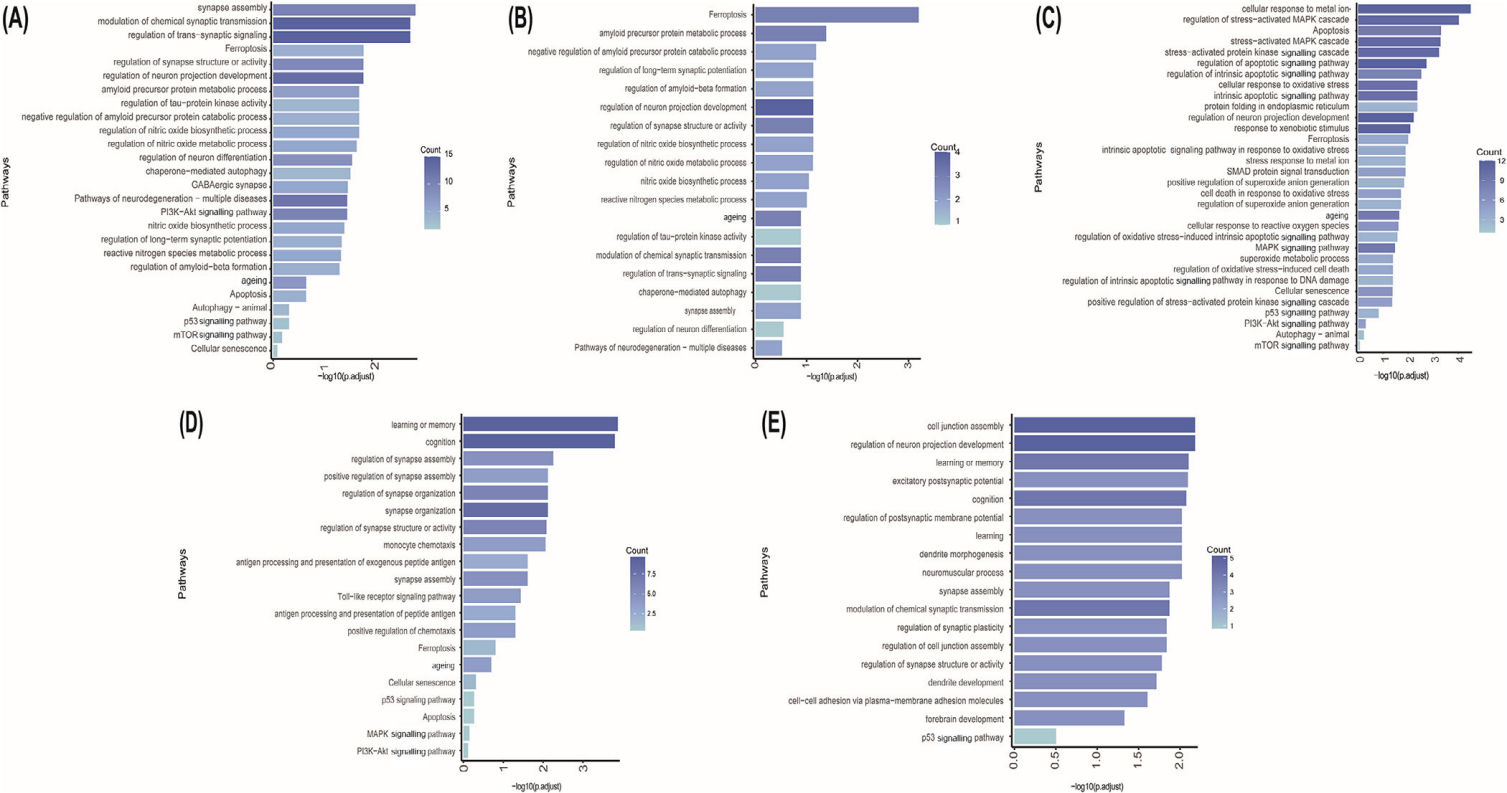
Functional enrichment analysis revealed distinct transcriptomic dysregulation in major cell types of focal cortical dysplasia (FCD). (A–E) Gene Ontology (GO) and Kyoto Encyclopedia of Genes and Genomes (KEGG) pathway enrichment analyses of differentially expressed genes (DEGs) identified from five principal neural populations: inhibitory neurons (A), excitatory neurons (B), astrocytes (C), microglia (D) and oligodendrocytes (E). Bar plots displayed the top enriched biological processes or signalling pathways ranked by significance (−log_10_ adjusted *p*‐value) and DEG count (colour scale).

In inhibitory neurons, GO and KEGG pathway enrichment analysis revealed significant involvement of the disease group in synaptic processes, ferroptosis, regulation of nitric oxide metabolism, regulation of neuron differentiation, GABAergic synapses and the PI3K‐Akt signalling pathway (Figure 2A). Consistently, GSEA demonstrated significant enrichment in pathways related to neurodegeneration, ageing and ferroptosis (Figure S3A). These results demonstrated that inhibitory neurons in FCD could be involved in synaptic processes, ferroptosis and ageing processes.

In excitatory neurons, GO and KEGG pathway enrichment analyses highlighted significant enrichment in pathways associated with ferroptosis and amyloid metabolism (Figure 2B). Furthermore, GSEA revealed that excitatory neurons in the FCD group exhibited upregulation of pathways related to neurodegenerative diseases, autophagy and amyloid precursor protein metabolism (Figure S3B). These findings highlight the potential role of ferroptosis and amyloid metabolism in the progression of disease in excitatory neurons.

In astrocytes, GO and KEGG pathway enrichment analyses revealed significant enrichment in pathways related to cellular response to mental ion, regulation of stress‐activated MAPK pathway, apoptosis, cellular response to oxidative stress, ferroptosis and ageing (Figure 2C). These results indicated that astrocytes in the disease group exhibited a strong oxidative stress state, accompanied by concurrent activation of apoptosis, ferroptosis and ageing. These findings were additionally confirmed by GSEA (Figure S3C).

In microglial cells, GO and KEGG pathway enrichment analyses identified significant activation of pathways associated with learning and memory, cognition, regulation of synapse assembly and toll‐like receptor signalling pathway (Figure 2D). These findings demonstrate a potential role of microglia in regulating synaptic pruning in the disease group. Additionally, GSEA revealed significant upregulation of the ferroptosis pathway (Figure S3D), highlighting the function of ferroptosis in microglial dysfunction.

In oligodendrocytes, pathways related to cell junction assembly, learning and memory, cognition and regulation of postsynaptic membrane potential were notably upregulated, as evidenced by GO and KEGG pathway enrichment analyses (Figure 2E). These results indicate the remarkable role of oligodendrocytes in the disease group, and the results were similarly attained through GSEA (Figure S3E).

Notably, across multiple cell types in FCD lesions, GO and KEGG pathway enrichment analyses, along with GSEA, consistently revealed significant upregulation of ferroptosis‐related pathways. This finding indicates that ferroptosis may play a central role in the pathogenesis of FCD across multiple cell types. Outcomes of GO and KEGG enrichment assays are provided in Tables S1A–E and S2A–E, respectively.

#### 3.3.1 Transcriptional characterization and pathway enrichment analysis of neuronal subclusters in FCD

**FIGURE 3:**
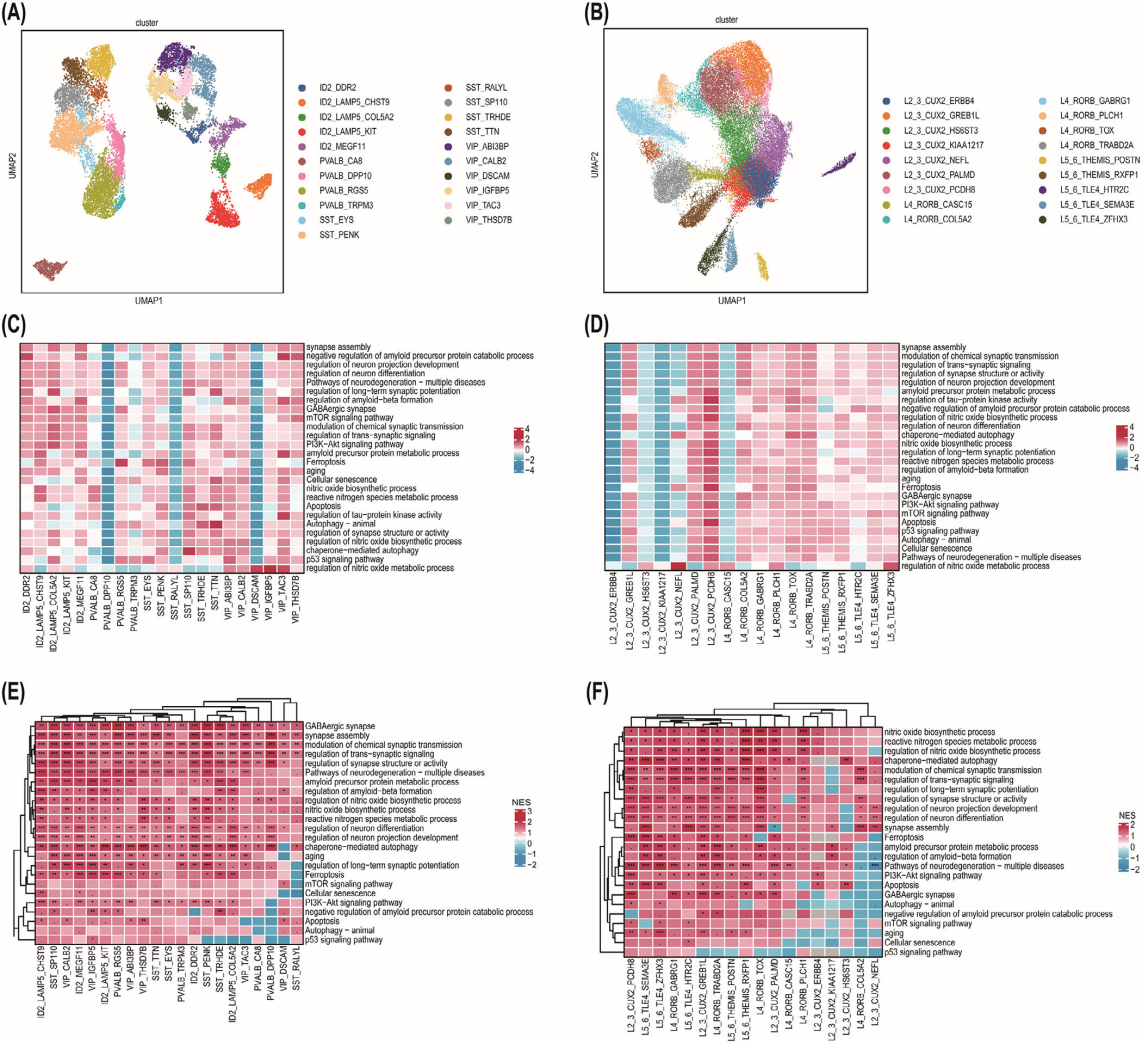
Single‐nuclei transcriptomic profiling revealed the heterogeneity of inhibitory and excitatory neurons in focal cortical dysplasia (FCD). (A and B) Uniform manifold approximation and projection (UMAP) visualization demonstrated disease‐associated subcluster evolution of inhibitory neurons (A) and excitatory neurons (B), with distinct compositional patterns distinguishing FCD from cortical controls. (C and D) Heat maps of UCell enrichment scores illustrating subcluster‐specific activation of selected biological processes and disease‐relevant pathways in inhibitory (C) and excitatory (D) neurons. (E and F) Gene set enrichment analysis (GSEA) heat maps displayed normalized enrichment scores (NES) for different inhibitory (E) and excitatory (F) neuron subclusters, with colour coding indicating selected gene ontology categories. Significance was denoted as *p < .05, **p < .01, ***p < .001.

To further explore the cellular heterogeneity identified through single‐nucleus transcriptomic profiling, neuronal populations were systematically deconvoluted into discrete subclusters based on canonical marker gene expression (Figure 3A,B and Figure S2B,C). Compositional analysis revealed quantitative differences in subcluster distributions between experimental cohorts (Figure S4A–E), whereas UCell scoring was used to elucidate their molecular signatures (Figure 3C,D). To explore functional mechanisms, GSEA scoring was carried out to identify pathway activation patterns specific to each subpopulation (Figure 3E,F). To address the issue of interpatient variability, UCell scores were precisely calculated utilizing a uniform gene signature. Subsequently, these scores underwent normalization in each sample. This strategic normalization step was specifically implemented to promote reliable and meaningful cross‐patient comparisons, thereby ensuring that the derived scores could be effectively juxtaposed across different patient cohorts. In the context of GSEA, the normalized enrichment scores (NES) inherently possess the capability to account for variations in gene set size and gene correlation structure. This intrinsic adjustment mechanism enhances the robustness of the NES, enabling more accurate and reliable comparisons across diverse samples. Consequently, the utilization of NES in GSEA not only mitigates the impact of interpatient variability but also enhances the overall reliability and validity of the comparative analysis.

##### 3.3.1.1 Inhibitory neurons

Inhibitory neurons were examined to characterize their molecular diversity and functional signatures. A total of 16 081 inhibitory neuron nuclei were stratified into 21 transcriptionally distinct subclusters using Leiden clustering and ontology‐informed annotation, guided by the expression of key marker genes, including *ID2*, *PVALB*, *SST* and *VIP* (Figure 3A). Nuclei from FCD types Ia, Ic, IIa and IIb were compared with controls (Figure S2B). Integration of datasets across subtypes and controls revealed broadly consistent subclustering, indicating robust alignment of cell populations. The relative proportions of major cell types were comparable across FCD subtypes and controls (Figure S4A).

UCell scoring indicated that most subclusters exhibited high enrichment scores for critical functions, including ageing, apoptosis, ferroptosis, GABAergic synapse, synapse assembly and trans‐synaptic signalling pathways. In contrast, subclusters, such as PVALB_DPP10, SST_RALYL and VIP_DSCAM, displayed a relatively lower enrichment for these pathways (Figure 3C). Consistently, GSEA scoring revealed significant enrichment of these pathways in most FCD subclusters compared with controls (Figure 3E). Detailed analysis indicated that abnormal activation of these pathways in numerous inhibitory neuronal subclusters could induce epileptogenesis. Notably, the ID2_LAMP5_COL5A2, PVALB_RGS5, SST_SP110 and VIP_IGFBP5 subclusters exhibited a significant upregulation of ageing, apoptosis and ferroptosis pathways, confirming their potential contribution to neuronal vulnerability and epileptogenesis in FCD. Moreover, in the majority of subclusters, FCD type Ic and FCD type IIa exhibited a more remarkable upregulation of the ferroptosis pathway compared with other types (Figure S5A).

##### 3.3.1.2 Excitatory neurons

A total of 44 810 excitatory neurons were characterized into 18 transcriptionally distinct subclusters using clustering and hierarchical annotation, guided by key marker genes, including *CUX2*, *RORB*, *THEMIS* and *TLE*. Subclusters were then compared across FCD subtypes and controls (Figure 3B and Figure S2C). Integration of FCD subtypes and the controls demonstrated general consistency across identified subclusters. Although the overall composition of excitatory neurons did not significantly differ between FCD and control groups, FCD IIa subtype exhibited a higher proportion of L2_3_CUX2_GREB1L neurons and a lower proportion of L2_3_CUX2_ERBB4 and L2_3_CUX2_KIAA1217 neurons compared with other FCD subtypes (Figure S4B).

UCell scoring indicated that most L5_6 subclusters had high scores in pathways related to ageing, apoptosis and ferroptosis, whereas several L2_3 subclusters achieved low scores, except for L2_3_CUX2_GREB1L, L2_3_CUX2_PALMD and L2_3_CUX2_PCDH8 (Figure 3D). GSEA scoring further revealed significant upregulation of ferroptosis, apoptosis and ageing pathways in most FCD subtypes, compared with controls, indicating their potential role in cortical dysplasia. Additionally, pathways, related to synapse assembly, trans‐synaptic signalling and nitric oxide metabolism, were also upregulated in most subclusters, suggesting their role in epileptogenesis (Figure 3F). Detailed analysis demonstrated that ferroptosis‐related pathways were upregulated in most subtypes across different subclusters, in which L2_3_CUX2_GREB1L and L2_3_CUX2_PCDH8 subclusters exhibited a significant upregulation across all FCD subtypes. Furthermore, nitric oxide metabolism was markedly enriched in most subclusters from FCD IIa and IIb subtypes, except for the L4_RORB_COL5A2 subcluster in FCD IIb, which exhibited a relatively lower enrichment (Figure S5B).

#### 3.3.2 Transcriptional characterization and pathway enrichment analysis of glial cell subclusters in FCD

The transcriptional landscape of glial cells in FCD was further explored through computational subclustering and pathway enrichment analyses. These analyses revealed the molecular diversity and potential functional roles of astrocytes, microglia and oligodendrocytes across different FCD subtypes.

##### 3.3.2.1 Astrocyte

**FIGURE 4:**
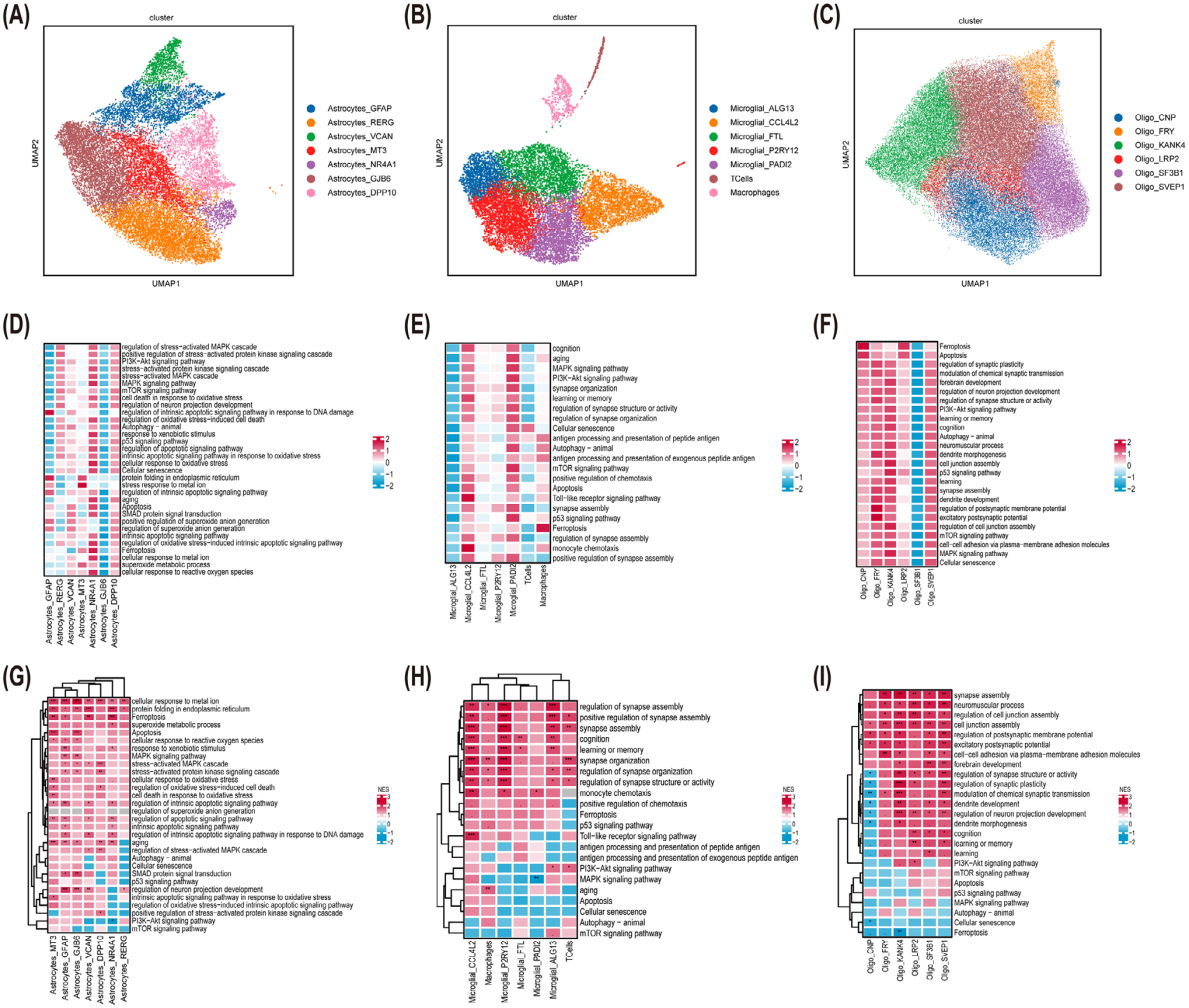
Single‐nuclei transcriptomic profiling revealed heterogeneity of astrocytes, microglia and oligodendrocytes in focal cortical dysplasia (FCD). (A–C) Comparative uniform manifold approximation and projection (UMAP) visualization demonstrated disease‐associated subclustering of astrocytes (A), microglia (B) and oligodendrocytes (C), highlighting distinct compositional patterns that differentiate FCD specimens from cortical controls. (D–F) Heat maps of UCell enrichment scores showing subcluster‐specific expression of selected biological processes and disease‐relevant pathways in astrocytes (D), microglia (E) and oligodendrocytes (F). (G–I) Gene set enrichment analysis (GSEA) heat maps of normalized enrichment scores (NES) for different subclusters of astrocytes (G), microglia (H) and oligodendrocytes (I). Each cell subcluster was colour‐coded to highlight selected gene ontologies. Significance was denoted as *p < .05, **p < .01, ***p < .001.

Clustering and hierarchical annotation of 17 868 astrocyte nuclei identified seven transcriptionally distinct subclusters (Figure 4A). Comparative analyses across FCD types Ia, Ic, IIa, IIb and controls demonstrated high concordance of subcluster identities, confirming robust integration of the datasets. Notably, FCD type IIb exhibited a significant enrichment of nuclei in *GFAP* subclusters (Figure S2D). Comparative analysis revealed a significant enrichment of RERG+ astrocytes in control specimens (42.21%) compared with FCD samples (26.49%, *p* < .001), a pattern that was conserved across all FCD subtypes (Figure 4D). Conversely, *GFAP* subclusters were significantly expanded in pathological specimens, particularly in FCD IIb, where they comprised 27.90% of the astrocyte population, indicating disease‐associated gliosis and cellular reorganization (Figure S4C).

UCell scoring highlighted that the *NR4A1* and *DPP10* subclusters exhibited high scores for BPs, including ferroptosis, ageing, apoptosis, oxidative stress response and metal ion homeostasis (Figure 4D). The results of GSEA further indicated a significant upregulation of pathways related to cellular response to metal ion and ferroptosis across most FCD subclusters compared with controls (Figure 4G). Notably, specific subclusters exhibited remarkable activation of the ferroptosis pathway, including the *GFAP* subcluster in FCD IIb subtype, *VCAN* subcluster in FCD IIa and IIb subtypes, *MT3* subcluster in FCD Ic and IIb subtypes, *NR4A1* subcluster in FCD Ia, Ic and IIb subtypes and the *DPP10* subcluster in FCD IIa subtype (Figure S5C). These findings demonstrate that oxidative stress‐induced ferroptosis may play a remarkable role in FCD progression, contributing to glial dysfunction and disease progression.

##### 3.3.2.2 Microglia

Subclustering of 12 482 microglial nuclei identified five transcriptionally distinct microglial subclusters, along with T cells and infiltrating macrophages (Figure 4B). Integration of FCD subtypes with control cortex demonstrated a consistent alignment of subcluster identities (Figure S2E). The composition and relative proportions of microglial subclusters did not significantly differ between FCD and control groups (Figure S4D).

UCell analysis revealed that subclusters, particularly *CCL4L2* and *PADI2*, had elevated activity in pathways related to learning, memory, synapse assembly and cognition. Additionally, well‐established FCD‐related mechanisms, such as the MAPK, PI3K‐AKT and mTOR signalling pathways, also demonstrated elevated activity scores in these subclusters. In contrast, these pathways showed significantly lower scores in the *ALG13* subclusters and T cells (Figure 4E). GSEA scoring demonstrated significant enrichment of these pathways in the majority of FCD subclusters compared with controls (Figure 4H). Subtype‐specific analysis indicated that microglia in FCD Ia showed consistent upregulation of pathways related to synapse remodelling and cognitive function across all five subclusters. In contrast, most microglial subclusters in the FCD Ic subtype demonstrated downregulation of synapse assembly processes, indicating divergent microglia functions across different disease subtypes. Notably, except for the *CCL4L2* and *PADI2* subclusters in FCD IIb, the majority of subclusters in the FCD II subtypes were upregulated in these pathways (Figure S5D).

##### 3.3.2.3 Oligodendrocytes

A total of 62 351 oligodendrocyte nuclei were classified into six distinct subclusters based on transcriptional profiles (Figure 4C, Figures S2F and S4E). UCell scoring indicated that, compared with the *SF3B1* subcluster, other subclusters mainly exhibited significantly higher scores in fundamental BPs, including ferroptosis, apoptosis, regulation of synapse structure or activity and synapse assembly (Figure 4F). GSEA further revealed significant enrichment of pathways related to synapse assembly, regulation of cell junction assembly and regulation of synapse structure or activity pathways in most FCD subclusters compared with the controls (Figure 4I). However, FCD Ic displayed a unique trend, involving downregulation of these pathways compared with controls, highlighting subtype‐specific oligodendrocyte responses (Figure S5E).

Collectively, the comprehensive analysis of glial cell subclusters in FCD revealed significant transcriptional and pathway alterations across astrocytes, microglia and oligodendrocytes. These findings highlighted the diverse roles of glial cells in FCD pathology, with particular emphasis on oxidative stress, ferroptosis and synaptic dysfunction. Further details regarding the GSEA results are presented in Table S3A–E, respectively.

### 3.4 Construction of gene regulatory networks in inhibitory neurons of FCD

To further elucidate the transcriptional mechanism underlying the development and dysfunction of inhibitory neurons in FCD, gene regulatory networks were constructed with a focus on TFs, modulating gene expression. It is noteworthy that the analysis concentrated on TFs and transcriptional regulatory networks. Accordingly, key ferroptosis executors, such as GPX4 and SLC7A11, were not prioritized, as their functional activity is predominantly regulated through post‑transcriptional and post‑translational mechanisms, including mRNA stability, protein turnover and enzymatic regulation, rather than by changes in transcript abundance, and therefore did not exhibit significant differential expression at the mRNA level in this dataset. Utilizing the single‐cell regulatory network inference and clustering (SCENIC) method, a total of 101 high‐confidence regulons were identified, involving 2720 target genes, with individual regulons comprising between 3 and 192 genes. These regulons provided a basis for dissecting core regulatory programs associated with neuronal identity and pathological transformation.

To establish cell‐type‐specific associations between regulons and inhibitory neurons across FCD subtypes, the top five specific regulons were analysed in different cell types using the RSS. A number of shared TF regulons were identified across inhibitory neurons from both FCD and control groups using RSS distribution (Figure S6A). These shared regulons may represent conserved transcriptional regulators contributing to the gene expression patterns identified in both disease and non‐disease states.

Utilizing ferroptosis‐related transcriptional regulators, regulon activity was visualized through heat maps. Several TFs previously linked to ferroptosis were significantly activated in inhibitory neurons of FCD compared with controls, and the strongest activation was found in the FCD Ic and IIa subtypes. Specifically, IRF9 expression level was upregulated in inhibitory neurons of FCD Ic, whereas NR2F2 showed a significant activation in FCD IIa. Both subtypes also exhibited elevated expression levels of RAD21, DLX1, NFE2L1, STAT1, FOSB and DLX2 (Figure S6B).

To assess the interconnectivity and modularity of TFs, pairwise similarity of regulon activity profiles was quantified across the dataset using the connection specificity index. This analysis grouped the 16 regulons into 4 major modules. Notably, ferroptosis‐related TF regulons, previously reported in other contexts, were clustered into a single coherent module, indicating coordinated regulatory activity in FCD (Figure S6C and Table S4). These results highlight the role of specific TF regulons in modulating ferroptosis and other key pathways in inhibitory neurons, implicating them in the molecular pathogenesis of FCD.

### 3.5 Identification of FRGs in FCD

**FIGURE 5:**
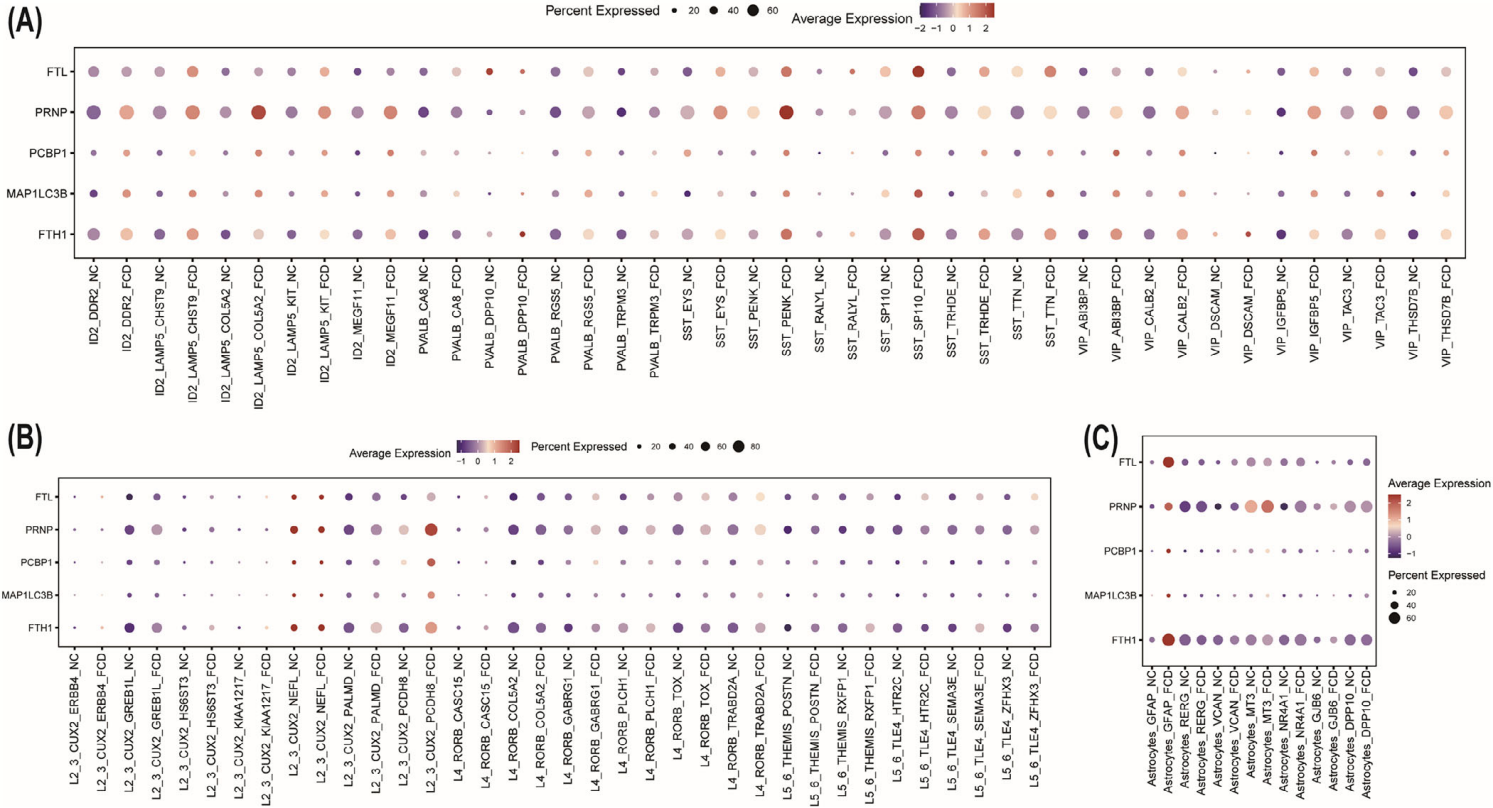
Expression levels of ferroptosis‐related genes in inhibitory neurons, excitatory neurons and astrocytes across focal cortical dysplasia (FCD) subtypes and controls. (A–C) Bubble plot depicting the expression levels of FRGs in inhibitory neurons (A), excitatory neurons (B) and astrocytes (C). Each row represented a subcluster from FCD subtypes or controls, whereas each column corresponded to a ferroptosis‐related gene. The colour intensity of the bubbles indicated the average expression level of each gene in a specific subtype relative to others, with darker shades representing higher expression levels. The size of each bubble reflected the percentage of cells in the subcluster expressing the indicated gene.

To identify FRGs in FCD, DEGs from inhibitory neurons, excitatory neurons and astrocytes identified through KEGG enrichment analysis were integrated with a manually curated list of known FRGs. This integrative approach pinpointed 5 key FRGs, involving ferritin light chain (*FTL*), ferritin heavy chain 1 (*FTH1*), poly rC binding protein 1 (*PCBP1*), prion protein‐encoding gene (*PRNP*) and microtubule‐associated protein 1 light chain 3B (*MAP1LC3B*) (Figure 5A–C and Table S5A–C).

The analysis revealed significant heterogeneity in the expression levels and cellular distribution of FRGs across FCD subtypes and controls, with a general trend of elevated expression levels of FRGs in FCD tissues. In inhibitory neurons, the expression levels of FRGs were consistently higher in all FCD subtypes compared with controls, with a notable enrichment in the ID2_LAMP5_COL5A2, SST_PENK and SST_SP110 subclusters (Figure 5A). The most remarkable alterations were identified in subtype‐specific subclusters, including ID2_LAMP5_COL5A2 subcluster in FCD IIc, SST_PENK subcluster in FCD IIa and SST_SP110 subcluster in FCD IIb. Moreover, FCD Ic and IIa exhibited a broader upregulation of FRGs across inhibitory neuron subclusters (Figure S7A).

In excitatory neurons, the L2_3_CUX2_PCDH8 subcluster in FCD Ic and FCD IIa demonstrated significantly higher expression levels of FRGs compared with controls (Figure 5B and Figure S7B). Similarly, in astrocytes, the GFAP subcluster in FCD exhibited markedly elevated expression levels of FRGs, particularly in the FCD IIb subtype (Figure 5C and Figure S7C).

These findings highlight the cell‐type‐ and subtype‐specific dysregulation of FRGs in FCD, demonstrating that these FRGs may play a pivotal role in the pathogenesis of FCD by modulating ferroptosis‐related pathways.

### 3.6 Validation of the expression levels of FRGs and biochemical markers in FCD

**FIGURE 6:**
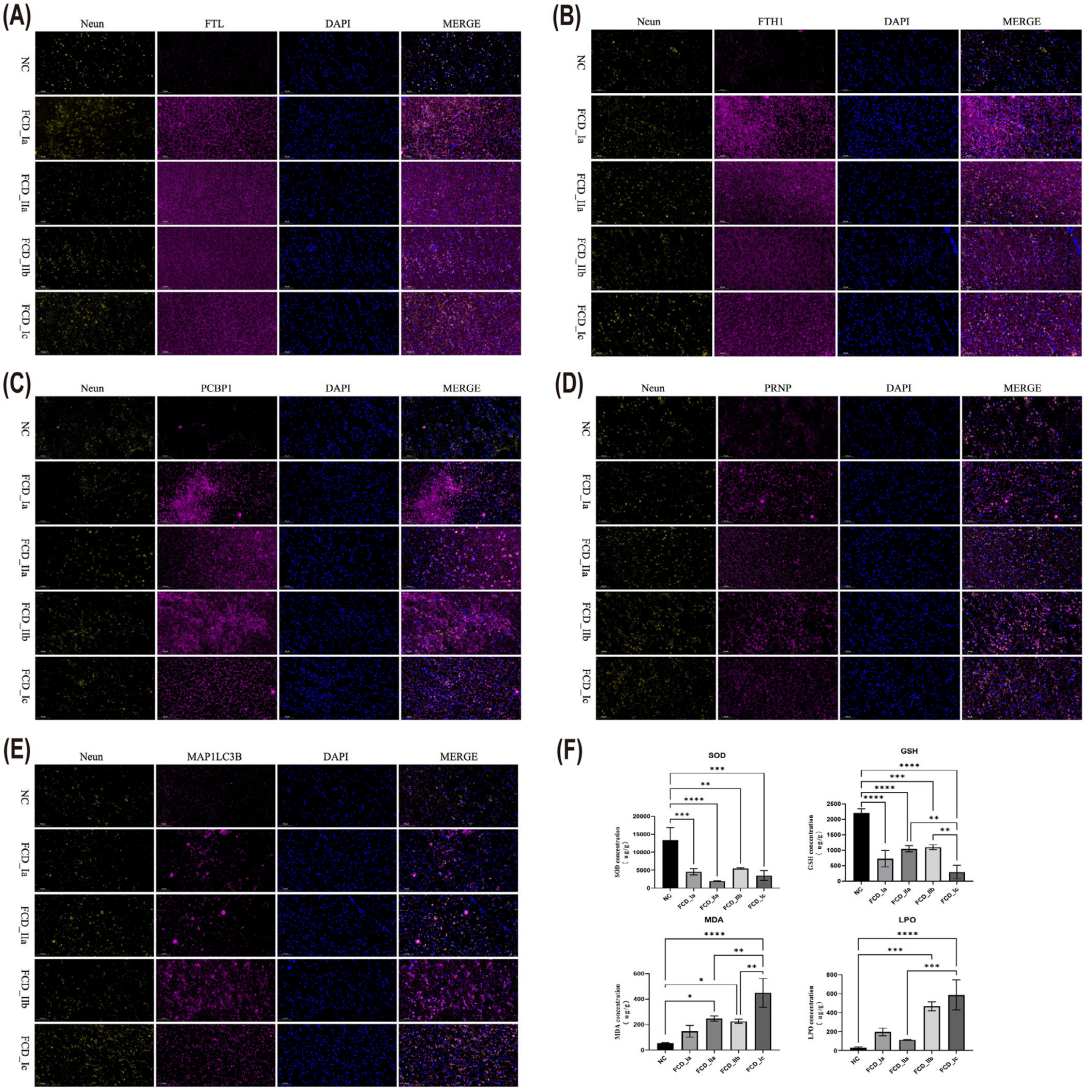
Immunofluorescence validation and biochemical assessment of ferroptosis‐related genes and lipid peroxidation markers in focal cortical dysplasia (FCD). (A–E) Representative immunofluorescence images of ferritin light chain (FTL) (A), ferritin heavy chain 1 (FTH1) (B), poly rC binding protein 1 (PCBP1) (C), prion protein‐encoding gene (PRNP) (D) and microtubule‐associated protein 1 light chain 3B (MAP1LC3B) (E) in the cortices of control and FCD subtypes. Immunostaining of neuronal nuclei (NeuN) (yellow), target proteins (pink) and DAPI (blue) showed the spatial distribution of cells in the FCD and controls. (F) Flow cytometry histograms showing the comparison of ferroptosis‐related biochemical markers between FCD subtypes and controls. GSH, glutathione; LPO, lipid peroxides; MDA, malondialdehyde; SOD, superoxide dismutase. *p < .05, **p < .01, ***p < .001, ****p < .0001.

To independently validate the upregulation of FRGs identified through transcriptomic analysis, immunofluorescence staining was conducted on formalin‐fixed, paraffin‐embedded cortical sections from FCD patients and controls. The results confirmed the upregulation of FTL, FTH1, PCBP1, MAP1LC3B and PRNP in the epileptogenic cortex of FCD patients compared with controls. Notably, FTL, FTH1 and PCBP1 exhibited particularly remarkable upregulation, as evidenced by robust immunolabeling with specific antibodies (Figure 6A–E).

Furthermore, biochemical analyses were carried out to assess the expression levels of key ferroptosis markers in FCD patients. The results demonstrated a significant decrease in the expression levels of SOD and GSH in FCD tissues compared with controls. Conversely, there was a significant elevation in the levels of MDA and LPO, which are indicators of lipid peroxidation and oxidative stress (Figure 6F). These findings indicated that ferroptosis has occurred in the FCD lesion area and supported the role of ferroptosis‐related pathways in the pathogenesis of FCD.

Taken together, these results provide compelling evidence for the upregulation of FRGs and the activation of ferroptosis‐related pathways in FCD, further implicating FRG dysregulation and ferroptosis in its pathogenesis.

### 3.7 CellChat analysis of intercellular communication in FCD

To systematically evaluate potential cell–cell signalling in the FCD microenvironment, intercellular communication analysis was performed using CellChat. This analysis encompassed major neural and non‐neural cell types identified in the snRNA‐seq data, including excitatory neurons, inhibitory neurons, astrocytes, microglia, oligodendrocytes, OPCs, ECs and fibroblasts. Comparative analysis between FCD and control tissues revealed that the overall number of inferred interactions was consistently high among excitatory neurons, inhibitory neurons and astrocytes in both groups, reflecting that these cell types form a core, active signalling network regardless of disease state (Figure S8A,B). This persistent, high‐intensity communication among principal neural cells indicates that the pathogenic contributions of highly dysregulated cell types, such as ECs and fibroblasts, are less likely to arise from the establishment of novel, direct signalling pathways with neurons and glia and instead occur through broader modulation of core network function and the surrounding microenvironment.

## 4 DISCUSSION

Notably, FCD subtypes I and II are among the most frequent pathological substrates of drug‐resistant epilepsy, whereas their cellular architecture and molecular mechanism remain incompletely defined. To address this gap, in the present study, snRNA‐seq was carried out on 24 brain tissues (18 FCD samples spanning subtypes Ia, Ic, IIa, IIb; 6 controls), generating the first high‐resolution transcriptomic atlas of FCD I/II. Integrative analysis identified 5 major cell types subdivided into 59 transcriptionally distinct subpopulations, highlighting the marked cellular heterogeneity of FCD. A recurrent molecular signature across both neuronal and glial compartments was robust activation of ferroptosis. Subtypes Ic and IIa exhibited more remarkable dysregulation of FRG patterns, particularly those linked to iron metabolism and oxidative stress, compared with other subtypes across most subclusters. These transcriptomic findings were confirmed by immunofluorescence and biochemical assays, which consistently demonstrated upregulation of FRGs and biomarkers, including elevated lipid peroxidation and depleted antioxidant reserves, in FCD relative to controls. Collectively, the findings demonstrated that ferroptosis may play a significant role or act as a concurrent mechanism in the pathogenesis of FCD. Although preliminary, the findings highlight the potential role of ferroptosis‐related pathways in the disease process. Targeting these pathways may provide a promising therapeutic strategy for further exploration.

### 4.1 Ferroptosis as a central pathogenic mechanism in FCD

Ferroptosis, an iron‐dependent, lipid peroxidation‐driven form of regulated cell death, has emerged as a critical regulator of neuronal vulnerability in neurological disorders.^26^, ^27^, ^28^ In epilepsy, disruption in iron homeostasis was well‐documented,^29^, ^30^ and pharmacological inhibition of ferroptosis has shown neuroprotective effects against seizure‐induced damage.^7^, ^28^ The snRNA‐seq analysis indicated a notable activation of ferroptosis‐related pathways across various major cell types in FCD, highlighting its potential role in disease progression.

Among them, inhibitory neurons displayed particularly robust activation of ferroptosis‐related pathways. This finding is mechanistically significant, as impaired inhibitory signalling is a hallmark of epileptogenesis. Previous research has reported that ferroptosis, characterized by iron‐dependent lipid peroxidation, could lead to neuronal cell death, thereby exacerbating excitatory‐inhibitory imbalance in cortical networks.^31^ The dysregulation of inhibitory neurons may be particularly relevant in FCD, where altered synaptic processes are implicated in seizure generation.

In excitatory neurons, co‐enrichment of ferroptosis and amyloid‐related gene patterns highlights a multifaceted role of oxidative stress in FCD progression. The interaction between ferroptosis and amyloidogenic process has been documented in neurodegenerative diseases, demonstrating that similar mechanisms may also contribute to FCD.^32^, ^33^ The upregulation of these pathways in excitatory neurons may promote both initiation and propagation of epileptiform activity in FCD.

Astrocytes, as metabolic and homeostatic support cells, also exhibited remarkable upregulation of ferroptosis and apoptosis pathways. This finding implies a state of heightened oxidative stress, indicating elevated oxidative stress. Previous studies have implicated reactive astrocytosis and glial ferroptosis in promoting neuroinflammation and secondary neuronal injury.^34^, ^35^ These findings demonstrate that astrocytes are not merely passive responders to neuronal injury but also are active contributors to the pathogenesis of FCD, potentially influencing disease progression through their metabolic and neuroprotective functions. Insights into ferroptosis‐related pathways across diverse cell types enhance understanding of the cellular mechanisms underlying FCD and highlight ferroptosis as a potential therapeutic target. Further studies will be required to assess the efficacy and clinical relevance of such strategies.

### 4.2 Ferroptosis activation in FCD‐specific cellular subclusters

Integrated GO/KEGG pathway enrichment analyses and subcluster‐level enrichment profiling revealed cell type–specific activation of ferroptosis‐related pathways in FCD. Among the five major brain cell populations examined, excitatory neurons, inhibitory neurons, astrocytes and microglia displayed a remarkable enrichment of ferroptosis‐related processes, whereas oligodendrocytes exhibited no significant alterations. Besides, the cell‐cell communication analysis revealed no significant quantitative differences in the overall strength or number of predicted ligand‐receptor interactions in ferroptosis‐related pathways between FCD and control tissues. This demonstrates that the core intercellular signalling architecture is largely preserved in FCD. This directs the mechanistic concentration toward cell‐intrinsic dysregulation, including elevated oxidative stress, altered iron metabolism or reduced antioxidant capacity in specific cell types, rather than a wholesale rewiring of paracrine signalling networks.

In inhibitory neurons, ferroptosis‐related pathways were more strongly upregulated in FCD types Ic and IIa than in other subtypes across most subclusters. Notably, the inhibitory neuron subcluster ID2_LAMP5_COL5A2 exhibited the most significant upregulation. The ID2_LAMP5_COL5A2 subcluster is implicated in synaptic modulation and network stability. It was thus hypothesized that ferroptosis could preferentially impair this specific inhibitory neuronal population. The vulnerability of these neurons to ferroptotic death or dysfunction could compromise their role in maintaining synaptic inhibition, thereby contributing directly to the disinhibition and cortical hyperexcitability that are hallmark features of FCD and its associated epilepsy. This provides a plausible cellular mechanism, linking subcluster‐specific molecular alterations to the circuit‐level pathophysiology of the disease. Furthermore, astrocyte subpopulations exhibited the most remarkable transcriptional activation, particularly in pathways related to reactive oxygen species metabolism, metal ion homeostasis and lipid peroxidation.

Mechanistically, ROS‐driven oxidative stress triggers lipid peroxidation and ferroptosis,^36^ whereas prolonged seizures further amplify ROS production, establishing a vicious cycle of ‘oxidative stress‐seizure propagation‐neuronal death’.^37^ Therapeutically, ROS modulation may concurrently suppress ferroptosis, control seizures and delay FCD progression. Biochemical assays confirmed the transcriptomic ferroptosis signature, exhibiting significantly elevated levels of lipid peroxidation markers (MDA and LPO) alongside depletion of antioxidant defences (SOD and GSH) in FCD tissues. Inhibitory neurons and astrocytes also exhibited upregulation of genes linked to cellular senescence and apoptosis, indicating a multifaceted cell‐death landscape in FCD. Collectively, these findings demonstrate that ferroptosis may play a notable role in the pathogenesis of FCD, providing a basis for exploring therapeutic strategies that incorporate ferroptosis inhibition. Such approaches may be further enhanced by combining ferroptosis modulation with interventions targeting oxidative stress, glial dysfunction or other cell death pathways.

Notably, the analysis revealed concurrent enrichment of both the mTOR and ferroptosis pathways in specific subclusters of inhibitory neurons, excitatory neurons and microglia in FCD. This co‐enrichment prompts a critical inquiry into their potential interaction, given the established role of mTOR pathway dysregulation as a cornerstone of FCD type II pathogenesis.

A bidirectional regulatory model was proposed that integrated ferroptosis into the known molecular landscape of FCD. On the forward arm, hyperactivation of mTORC1, a hallmark of FCD II, was found to significantly lower the cellular threshold of ferroptosis through two synergistic mechanisms^38^: (1) an iron‐import axis, where mTORC1‐STAT3 signalling upregulated iron transporters (*TFRC*, *STEAP3* and *SLC39A14*), increasing cytosolic Fe^2+^ level to fuel the Fenton reaction; and (2) a lipid‐remodelling axis, where mTORC1‐SREBP‐1c enhanced *SCD1* activity and stabilized *ACSL4*, thereby enriching cellular membranes with peroxidation‐prone polyunsaturated fatty acids (e.g., arachidonic and adrenic acids). Conversely, on the feedback arm, the build‐up of ferroptotic stress (e.g., GSH depletion and the accumulation of lipid peroxidation product 15‐HpETE‐PE) has been shown to inhibit mTORC1 activity via the GATOR1‐Rag and AMPK‐TSC2 signalling pathways, potentially forming a compensatory brake.^39^

This integrative framework not only positions ferroptosis as a consequential downstream component of mTOR‐driven pathology but also presents a novel therapeutic rationale. Combining mTOR inhibitors (e.g., everolimus) with ferroptosis blockers (e.g., liproxstatin‑1) could synergistically control seizures while mitigating iron‐mediated neurotoxicity, a strategy meriting further preclinical investigation.

### 4.3 Key FRGs in FCD pathogenesis

In this study, five FRGs (*FTL*, *FTH1*, *PCBP1*, *PRNP* and *MAP1LC3B*) were identified as key contributors to the pathophysiology of FCD. These genes exhibited differential expression across both neuronal and glial populations and were validated by immunofluorescence and transcriptomic profiling, implicating them in ferroptosis activation and FCD‐specific molecular dysregulation.

Among the five key FRGs identified, *FTL* plays a crucial role in iron metabolism and cellular protection against oxidative stress. The molecular basis underlying *FTL*‐mediated regulation of ferroptosis is complex. On one hand, by binding to free iron, *FTL* reduces intracellular labile iron content, thereby attenuating iron‐catalysed ROS generation and oxidative damage to suppress ferroptosis. Conversely, insufficient *FTL* expression impairs iron storage capacity, sensitizing cells to ferroptotic death. On the other hand, *FTL* overexpression may trigger a phenomenon often described as the ‘iron paradox’, potentially disrupting cellular iron homeostasis and promoting the release of redox‐active iron.^40^ This liberated catalytic iron accelerates lipid peroxidation, leading to accumulation of lipid peroxidation products and subsequent induction of ferroptosis.^41^ This dual regulatory mechanism highlights the complexity and multifaceted nature of *FTL* in modulating ferroptosis. This finding is consistent with previous studies that have highlighted the role of iron dysregulation in neurodegenerative diseases, indicating that *FTL* may be relevant to mitigating ferroptosis‐related neuronal damage in FCD.^31^

*FTH1*, which catalyses the oxidation of Fe^2+^ to Fe^3+^, complements the function of *FTL* in iron homeostasis. It is integral to iron storage and homeostasis, and its expression level is mainly upregulated in response to oxidative stress.^42^ The significant upregulation of *FTH1* in FCD tissues highlights its potential role in the disease pathophysiology. An elevated *FTH1* expression level may represent an adaptive response to mitigate iron‐induced oxidative stress. Its close association with ferroptosis‐related pathways demonstrates that *FTH1* can modulate ferroptotic activity in FCD. Targeting *FTH1*‐dependent mechanisms may thus represent a potential strategy to influence ferroptosis and improve clinical outcomes, although further validation is required.

Notably, *PCBP1* functions as both an iron chaperone and an RNA‐binding regulator, playing a role in iron metabolism and RNA binding. *PCBP1* enhances the uptake of iron and regulates the expression levels of various genes involved in iron homeostasis.^43^ The upregulation of *PCBP1* in FCD indicates dysregulation of iron metabolism that may contribute to the pathogenesis of the disease. Given its multifaceted role in cellular iron management and its association with ferroptosis, *PCBP1* may represent a critical node in the network of molecular events, leading to neuronal injury in FCD. Future studies should elucidate the precise mechanisms by which *PCBP1* influences ferroptosis, thereby providing valuable insights into potential therapeutic strategies for FCD.

The identification and validation of *FTL*, *FTH1* and *PCBP1* as key FRGs in FCD demonstrate that ferroptosis may play a role in the disease's pathogenesis. These findings advance understanding of the molecular mechanisms underlying FCD and highlight ferroptosis modulation as a potential strategy to mitigate neuronal injury. Further studies are required to delineate the functional roles of these FRGs and to evaluate their utility as biomarkers for disease progression and treatment response in FCD.

According to snRNA‐seq findings and prior evidence, *FTL* was proposed as a core driver gene contributing to ferroptosis in FCD. This prioritization was supported by its consistent and prominent dysregulation across multiple neural cell types in the snRNA‐seq data, accompanied by its well‐established central role in cellular iron metabolism, which is a process fundamental to ferroptosis. The mechanistic hypothesis indicated that *FTL* upregulation disrupted neuronal iron homeostasis, leading to iron overload, ACSL4‐mediated lipid peroxidation and functional impairment of GPX4, thereby triggering ferroptotic cell death and ultimately facilitating neuronal network hyperexcitability.

To functionally validate this hypothesis, a targeted in vitro strategy was designed. This involved knocking down *FTL* in neuronal models to reproduce the pro‐ferroptotic phenotype (assessed via cell viability, lipid peroxidation and mitochondrial integrity) and employing specific pharmacological rescue (e.g., ferrostatin‐1) to confirm the causal role of the ferroptosis pathway. Successful validation would not only solidify the pathogenic role of *FTL* but also highlight ferroptosis inhibition as a potential therapeutic direction for FCD.

Importantly, the computational predictions from this study, including the prioritization of *FTL*, provided a strong basis for generating mechanistic hypotheses. Although the targeted experimental strategy outlined above represents an important initial step, future studies employing approaches such as spatial transcriptomics—to validate intercellular interactions and marker expression in situ—and electrophysiological analyses—to directly assess the impact of ferroptosis on neuronal excitability—will be invaluable for achieving comprehensive and direct functional validation in the complex tissue microenvironment of FCD.

### 4.4 Targeting ferroptosis: A potential therapeutic direction for FCD

Current management of FCD remains largely symptomatic, relying primarily on antiepileptic drug therapy alongside supportive cognitive or neurological rehabilitation.^40^ Nevertheless, a remarkable proportion of patients develop pharmacoresistant epilepsy, rendering surgical resection the primary and often most effective treatment option for this population.^44^, ^45^ Despite surgical efficacy, a subset of patients is ineligible for resection or continue to experience seizures postoperatively, highlighting the need for mechanism‐based, non‐surgical therapeutic strategies. In this context, the present findings indicate that ferroptosis represents a biologically plausible and potentially modifiable pathway in FCD‐associated epileptogenesis. Supporting evidence from prior studies demonstrates that modulation of ferroptosis and iron metabolism can attenuate neuronal injury and epileptiform activity: GPX4 dysfunction promotes iron accumulation and lipid peroxidation, whereas ferroptosis inhibitors, such as liproxstatin‐1^46^ and ferrostatin‐1,^47^ reduce seizure‐associated neurodegeneration. In addition, iron chelation with deferoxamine has shown to suppress refractory post‐stroke epilepsy through regulation of transferrin‐mediated iron homeostasis.^48^ Collectively, these results position the FRGs identified in this study as promising candidates for therapeutic exploration, particularly for patients who are not surgical candidates or exhibit persistent seizures after resection.

Building on this mechanistic framework, the present findings nominate ferroptosis as an actionable therapeutic target in FCD and provide a rationale for a staged translational strategy. Initial studies should evaluate the efficacy of established ferroptosis inhibitors (e.g., ferrostatin‐1 and liproxstatin‐1) in patient‐relevant in vitro models, including neural progenitor cells or cortical organoids carrying FCD‐associated mTOR pathway mutations. These investigations could be followed by in vivo proof‐of‐concept studies in rodent models of cortical malformation to assess therapeutic impact across multiple outcome measures, including seizure burden, histopathological preservation and modulation of ferroptosis biomarkers. In parallel, the repurposing of clinically available agents, such as iron chelators (e.g., deferiprone), may provide an expedited translational pathway. Collectively, these approaches provide a conceptually and experimentally grounded roadmap for developing novel interventions aimed at mitigating ferroptosis‐driven neuronal vulnerability in FCD.

Future research should explore the potential of these FRGs as biomarkers and therapeutic targets, with the aim of developing precision therapies targeting ferroptosis to advance personalized management of FCD.

### 4.5 Potential confounding effects of antiseizure medications (ASMs)

When interpreting the findings, the potential confounding effects of preoperative antiseizure medications (ASMs) must be considered. The ASM regimens in this cohort were highly heterogeneous, as detailed in Table 1, reflecting the typical management of pharmacoresistant epilepsy. No individual drug or treatment regimen was uniquely associated with any specific FCD subtype. This widespread, non‐systematic medication use makes a uniform drug effect an unlikely explanation for the coherent, subtype‐associated FRG expression patterns. Furthermore, existing literature demonstrates that several common ASMs, including valproate, lacosamide, levetiracetam, lamotrigine and oxcarbazepine, are generally associated with reducing oxidative stress and may inhibit ferroptosis.^49^, ^50^, ^51^, ^52^, ^53^, ^54^, ^55^, ^56^ Therefore, the significant upregulation of ferroptosis‐related signatures in FCD tissue, despite the prevalent use of such medications, represents a conservative and robust finding. This apparent paradox, where exposure to potentially inhibitory medications coexists with a robust pro‐ferroptotic signal, further supports the interpretation that the FCD disease process itself elicits a level of oxidative stress and ferroptotic burden sufficiently robust to outweigh the effects of non‐systemic pharmacological interventions, thereby highlighting the central role of this pathway in FCD pathology.

### 4.6 Limitations

Despite its strengths, this study has several limitations that should be acknowledged. First, although the sample size was sufficient to identify preliminary transcriptomic patterns, it might not fully capture the heterogeneity of FCD, particularly for subtype Ib, where limited availability of high‑quality tissue constrained generalizability. In addition, the analysis was restricted to FCD types I and II; exclusion of FCD type III due to its dual pathology and substantial confounding effects could limit extrapolation. Second, given the histopathological gradient from lesion core to perilesional regions, control tissues might retain residual epileptogenic activity, potentially confounding comparative analyses. Third, cell subpopulation identities were inferred primarily from marker gene expression without direct experimental validation, rendering these assignments hypothesis‑generating and necessitating confirmation using spatial transcriptomics or electrophysiological approaches. Fourth, although robust FRG signatures were identified through integrative analyses, in vivo functional validation remains lacking; targeted genetic perturbation in relevant neural models may be required to establish causal roles in FCD pathogenesis. Fifth, regulatory network inferences were based on transcriptional co‑expression and bioinformatic modelling and therefore did not constitute definitive evidence of direct transcriptional regulation, highlighting the need for experimental validation, including ChIP‑qPCR or luciferase reporter assays. Sixth, the cross‑sectional study design might limit inference regarding temporal and causal relationships, including the timing of ferroptosis activation and its association with postoperative seizure outcomes, highlighting the need for longitudinal and prospective studies. Finally, prior exposure to ASMs was not systematically controlled and might influence transcriptional profiles, and the exclusive inclusion of participants of Chinese ethnicity might limit generalizability to other populations.

## 5 CONCLUSIONS

In conclusion, this study provided a high‐resolution single‐nucleus transcriptomic atlas of FCD subtypes I and II, implicating ferroptosis as a potential pathogenic mechanism across multiple neural lineages. Single‐nucleus transcriptomic profiling delineated distinct cellular subtypes and their ferroptosis‐related transcriptional programs, demonstrating consistent upregulation of ferroptosis‐related pathways and oxidative stress signatures that may underlie cell‐type‐specific dysfunction in FCD. This dataset constitutes a reference resource for dissecting cellular heterogeneity in cortical malformations and provides mechanistic insights into the molecular pathology of FCD. In addition, the identification of ferroptosis‐related molecular signatures nominates potential biomarkers and therapeutic targets that warrant systematic validation in the context of epilepsy and cortical dysplasia.

## AUTHOR CONTRIBUTIONS

Dongfang Zou conceived and designed the study, supervised the project, performed data analysis and critically revised the manuscript. Qingyang Zeng and Zhiqiang Lin collected and curated the clinical data. Qingyang Zeng performed the data analysis and drafted the initial manuscript. Fengjun Zhu and Dezhi Cao coordinated patient recruitment and contributed to clinical data interpretation. Lin Li, Zeshi Tan, Cong Li, Xiaofan Ren and Yidi Liu assisted with patient enrolment and were responsible for obtaining surgical specimens. All authors have read and approved the final manuscript.

## CONFLICT OF INTEREST STATEMENT

The authors declare no conflicts of interest.

## ETHICS STATEMENT

This study was approved by the Medical Ethics Committee of the Shenzhen Children's Hospital (Approval no.202308102), and informed consent was obtained from the parents or legal guardians of all participants.

## Supporting information



Supporting Information

Supporting Information

## Data Availability

The raw single‐nucleus RNA sequencing data generated in this study have been deposited in the NCBI Sequence Read Archive (SRA) under the accession number of PRJNA1336443 and are publicly accessible at https://www.ncbi.nlm.nih.gov/sra/PRJNA1336443. All processed and structured analysis datasets supporting the findings of this study and used to generate the figures and conclusions have been curated and deposited in the public repository Figshare (https://figshare.com/) under the permanent DOI: https://doi.org/10.6084/m9.figshare.30988093.
