# Supplementary material for: Single‐nucleus RNA sequencing reveals ferroptosis as a potential contributor to the pathogenesis of focal cortical dysplasia
Source: Clin Transl Med. 2026 Apr 29;16(5):e70668. doi: 10.1002/ctm2.70668 (PMC13125963; doi:10.1002/ctm2.70668)
Supplement: Supplementary file 1 — Supporting Information [file CTM2-16-e70668-s001.docx]

**SUPPLEMENTARY MATERIALS**

**for**

**Single-Nucleus RNA Sequencing Reveals Ferroptosis as a Potential Contributor to the Pathogenesis of Focal Cortical Dysplasia**

Qingyang Zeng^1,2^, Fengjun Zhu^1^ Dezhi Cao^1^, Yang Sun^1^, Lin Li^1^, Zeshi Tan^1^, Cong Li^1^, Xiaofan Ren^1^, Yidi Liu^1^, Zhiqiang Lin^1^, Dongfang Zou^1^*

^1^ Epilepsy Center and Department of Neurology, Affiliated Shenzhen Children's Hospital of Shantou University Medical College, Shenzhen 518000, Guangdong, China;

^2^ Shenzhen Pediatrics Institute of Shantou University Medical College, Shenzhen 518000, Guangdong, China

* **Correspondence**

**Dongfang Zou** - Epilepsy Center and Department of Neurology, Affiliated Shenzhen Children's Hospital of Shantou University Medical College, Shenzhen 518000, Guangdong, China; https://org/0000-0001-6726-8704; Phone: +86-18938690738; Email: fiesta_zou@163.com

Qingyang Zeng, Fengjun Zhu, and Dezhi Cao contributed equally to this study.

**TABLE OF CONTENTS**

**SUPPLEMENTARY FIGURES ..........................................................................................................10**

SUPPLEMENTARY FIGURE 1..........................................................................................................2

SUPPLEMENTARY FIGURE 2..........................................................................................................3

SUPPLEMENTARY FIGURE 3........................................................................................................4-5

SUPPLEMENTARY FIGURE 4..........................................................................................................6

SUPPLEMENTARY FIGURE 5..........................................................................................................7

SUPPLEMENTARY FIGURE 6..........................................................................................................8

SUPPLEMENTARY FIGURE 7..........................................................................................................9

SUPPLEMENTARY FIGURE 8........................................................................................................10

**SUPPLEMENTARY TABLES ............................................................................APPENDIX .XLSX**

**
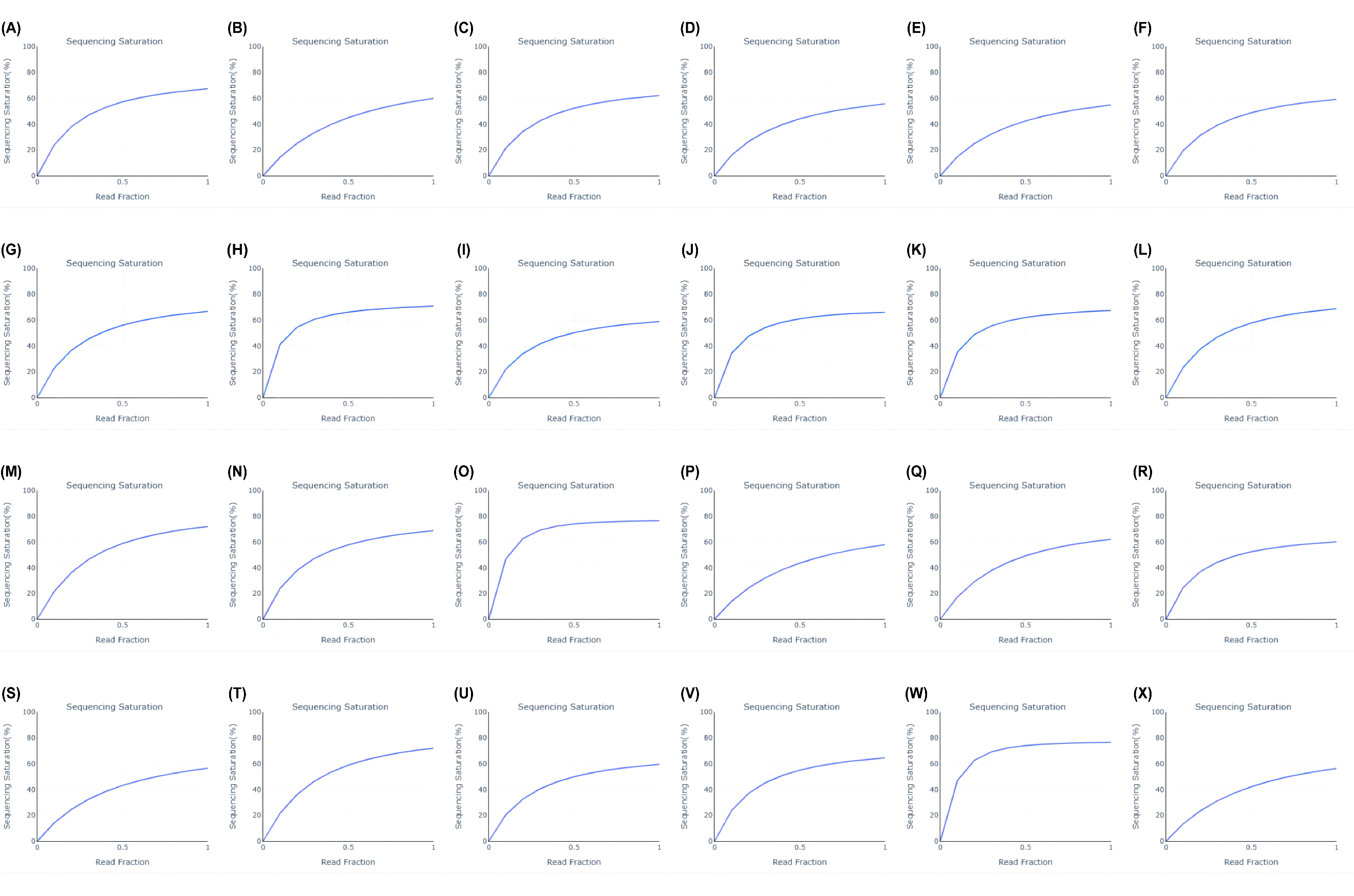
**

**SUPPLEMENTARY FIGURE 1. Sequencing saturation curves for the 24 brain tissue samples.** (A-R) Sequencing saturation curves of 18 highly epileptogenic cortical tissues with FCD. (S-X) Sequencing saturation curves of epileptogenic perilesional tissues with normal pathology (n=6). The sequencing saturation curve reached a plateau, indicating that the sampling depth was sufficient to comprehensively capture the transcriptomic diversity of the sample.





**SUPPLEMENTARY FIGURE 2.** **UMAP visualization of neuronal and non-neuronal subclusters with cell type annotation in FCD subtypes and normal controls (NC).** (A) UMAP analysis identified eight distinct cell types in FCD subtypes and NC, highlighting the subtype-specific cellular distributions across the groups. (B-F) Sub-clustering of major cell types across NC, FCD types Ia, Ic, IIa, and IIb. UMAP plots displayed inhibitory neurons (B), excitatory neurons (C), astrocytes (D), microglia (E), and oligodendrocytes (F) from the snRNA-seq datasets, illustrating the cellular heterogeneity and compositional differences across the groups.


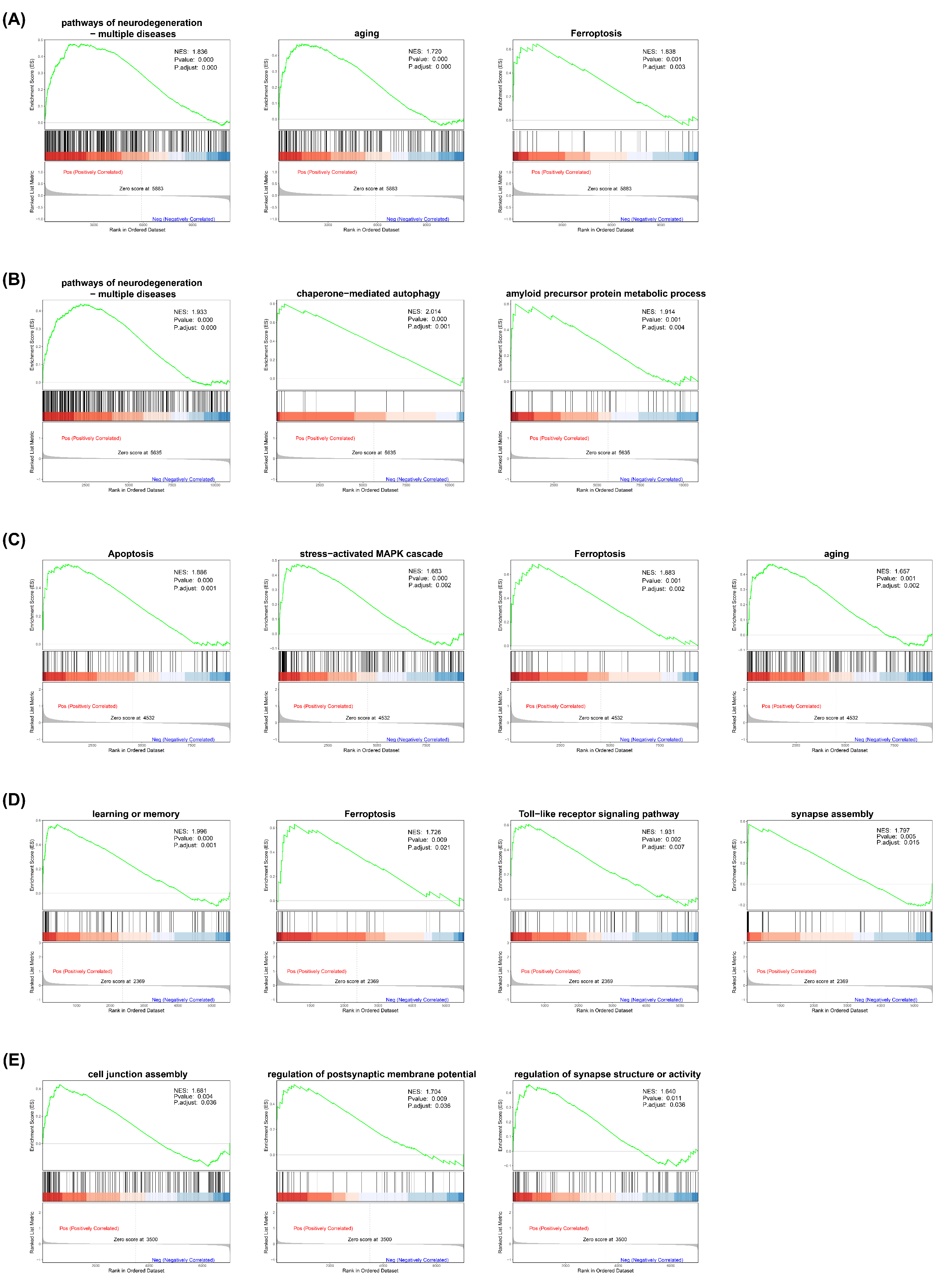


**SUPPLEMENTARY FIGURE 3. GSEA of major cell types revealed activation of subtype-specific pathways in FCD.** (A-E) Representative GSEA plots illustrating the enrichment of hallmark biological pathways across five distinct neural cell populations among inhibitory neurons (A), excitatory neurons (B), astrocytes (C), microglia (D), and oligodendrocytes (E) in FCD versus normal controls (NC). Representative examples included ferroptosis in inhibitory neurons, astrocytes, and microglia, highlighting distinct pathogenic processes across different cell populations in the FCD microenvironment. In each plot, the running enrichment score (green curve) was plotted against the rank-ordered gene list, with the peak indicating the core enrichment region of the gene set. Each panel displayed NES profiles, with the ranked gene sets plotted along the x-axis and running enrichment scores on the y-axis.


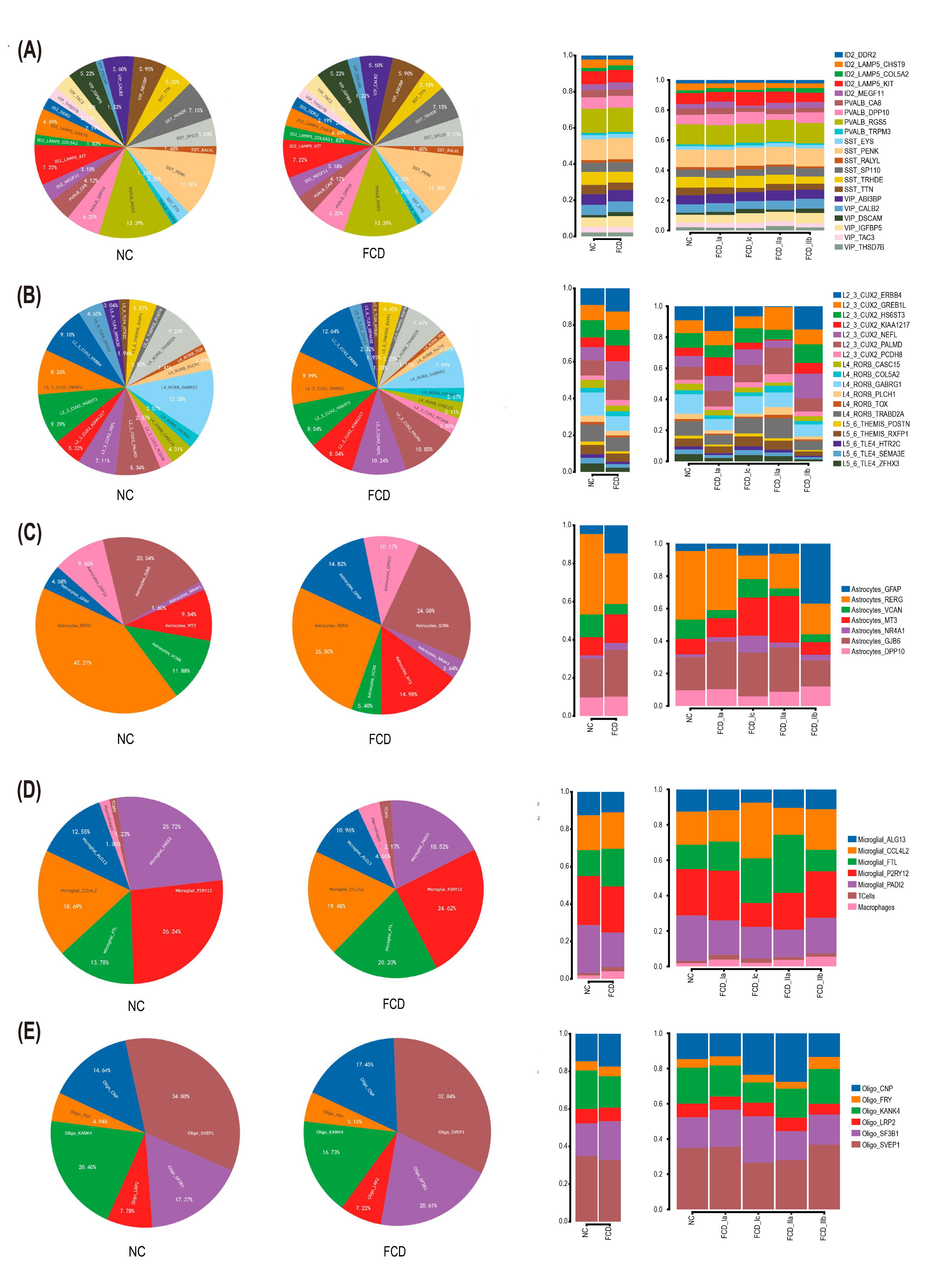


**SUPPLEMENTARY FIGURE 4. Cell-type-specific subcluster composition revealed subtype-associated alterations in FCD and proportional changes in FCD and normal controls (NC).** (A–E) Proportional distribution of cellular subclusters for inhibitory neurons (A), excitatory neurons (B), astrocytes (C), microglia (D), and oligodendrocytes (E) in FCD versus NC. Pie charts (with labeled percentages) and corresponding bar plots could visualize the relative abundance of each subcluster in the respective major cell type. These visualizations highlighted specific subclusters that were expanded or diminished in particular FCD subtypes, reflecting subtype-selective alterations in cellular states or populations in the FCD microenvironment.


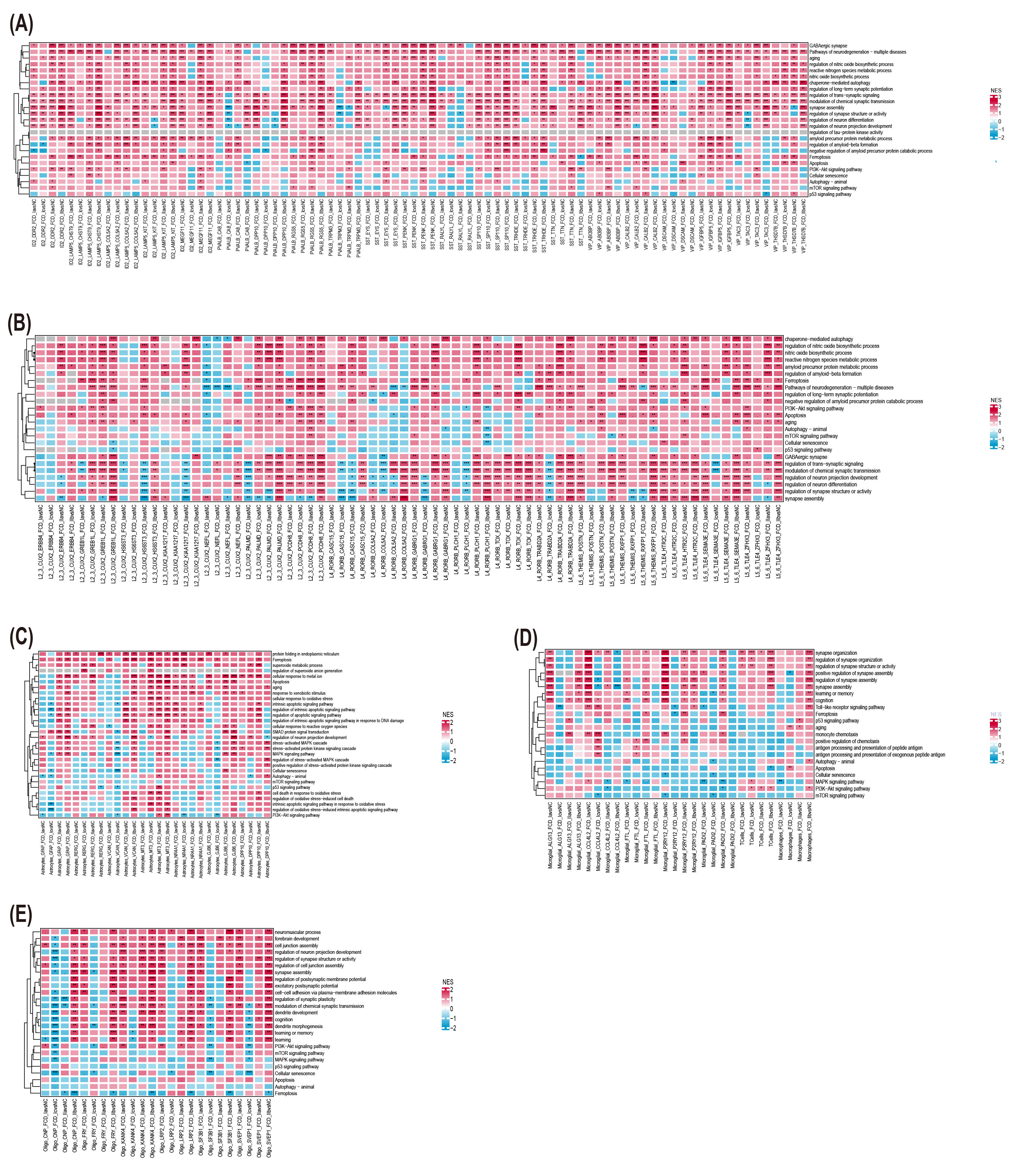


**SUPPLEMENTARY FIGURE 5. Subcluster-specific pathway enrichment patterns revealed by GSEA across major neural and glial cell types in FCD.** (A-E) Heatmaps, displaying NES from GSEA across cellular subclusters of inhibitory neurons (A), excitatory neurons (B), astrocytes (C), microglia (D), and oligodendrocytes (E). Each row represented a significantly enriched biological pathway or Gene Ontology (GO) term, while each column corresponded to a defined cellular subcluster. The analysis demonstrated that distinct pathogenic signaling pathways were selectively activated in specific subpopulations of each major cell type, highlighting the functional heterogeneity and subtype‑specific contributions of neural and glial cells in the FCD microenvironment. Statistical significance was denoted as follows: **p* < 0.05, ***p* < 0.01, ****p* < 0.001.


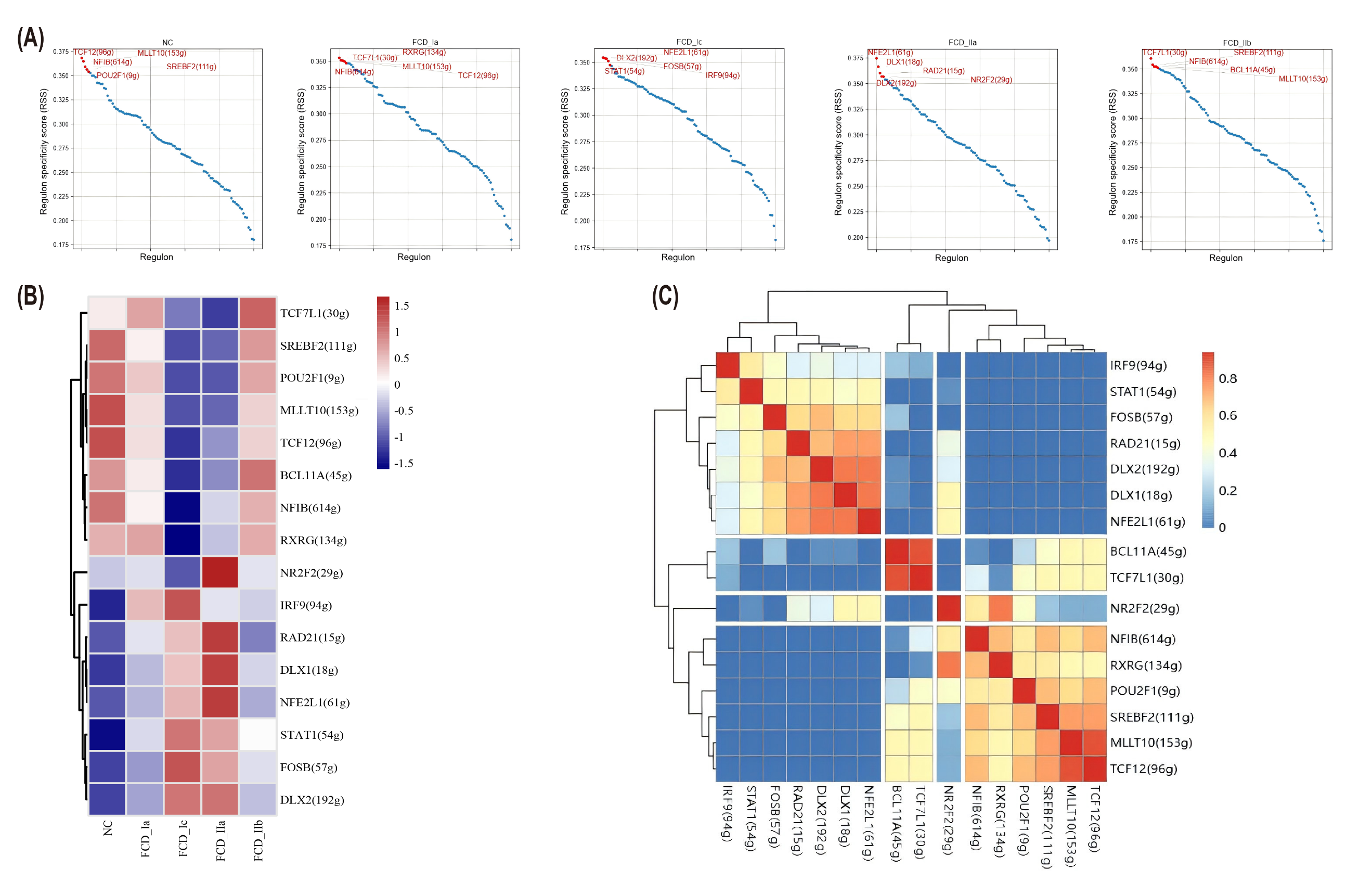


**SUPPLEMENTARY FIGURE 6. Transcriptional regulatory network analysis revealed subtype-specific regulon activity in FCD inhibitory neurons.** (A) Regulon Specificity Score (RSS) scatter plots for inhibitory neurons in normal controls (NC) and FCD subtypes (Ia, Ic, IIa, and IIb). The top five subtype-enriched transcription factor (TF) regulons in each group were labeled in red, identifying key TFs that could drive subtype-distinct transcriptional programs. The x-axis ranked all regulons based on RSS values, while the y-axis indicated RSS magnitude. (B) Heatmap depicting the activity patterns of selected TF regulons across NC and FCD subtypes. This visualization revealed clusters of regulons with coordinated activation or suppression in specific subtypes, indicating shared regulatory modules underlying pathological neuronal states. Note: The analysis concentrated on TFs and transcriptional regulatory networks. Accordingly, key ferroptosis executors, such as GPX4 and SLC7A11, were not prioritized, as their functional activity was predominantly regulated through post‑transcriptional and post‑translational mechanisms, and therefore did not exhibit significant differential expression at the mRNA level in this dataset. (C) The interconnectivity and modular organization of TFs were evaluated by quantifying pairwise similarities among their regulon activity profiles across the full dataset using the Connection Specificity Index (CSI). This analysis grouped 16 regulons into 4 major modules.

**
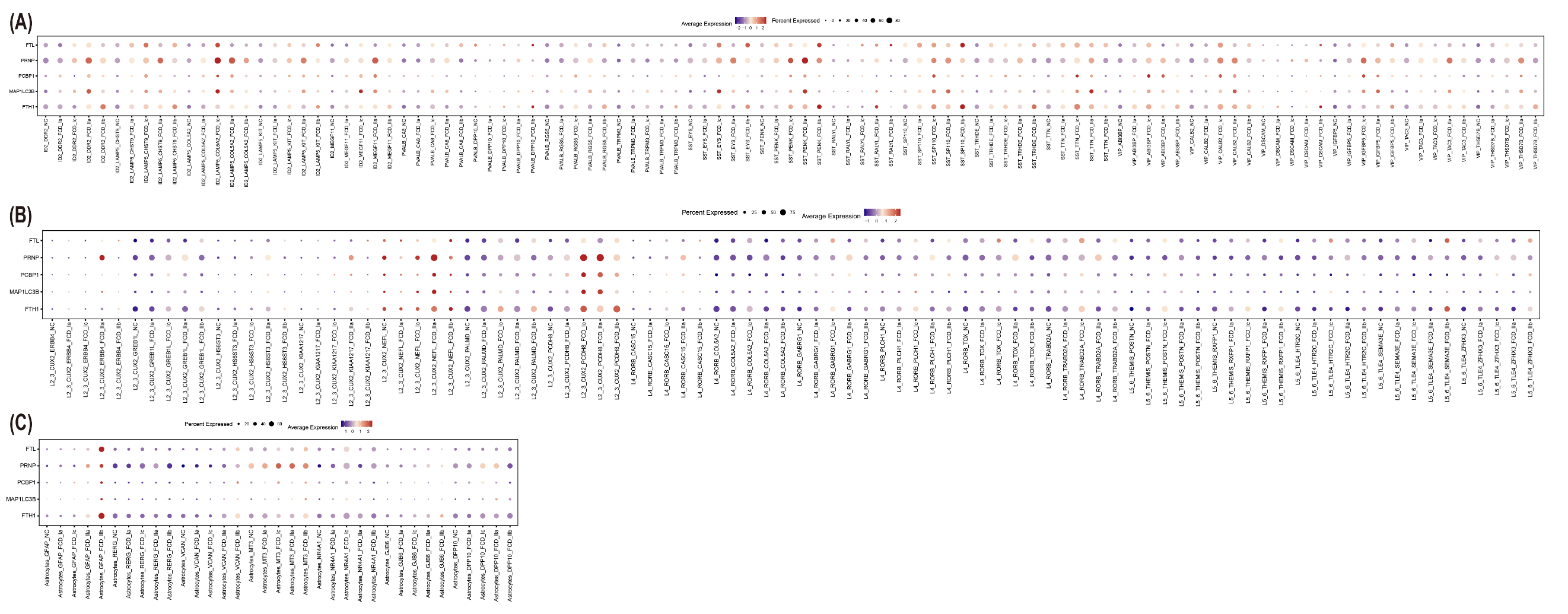
**

**SUPPLEMENTARY FIGURE 7. Subcluster-specific expression levels of ferroptosis-related genes (FRGs) in FCD subtypes and normal controls (NC).** (A-C) Dot plots illustrated the expression levels of selected FRGs across subclusters of inhibitory neurons (A), excitatory neurons (B), and astrocytes (C), respectively, in FCD subtypes and NC. Each row represented a cellular subcluster, and each column corresponded to an individual FRG. The color gradient reflected the average normalized expression level in each subcluster, while dot size indicated the proportion of cells expressing the gene. The size of each bubble reflected the percentage of cells in the subcluster expressing the indicated gene. The analysis revealed that five key FRGs were differentially expressed across FCD cell subclusters, including ferritin light chain (FTL), ferritin heavy chain 1 (FTH1), poly rC binding protein 1 (PCBP1), the prion protein-encoding gene (PRNP), and microtubule-associated protein 1 light chain 3 (MAP1LC3/LC3).


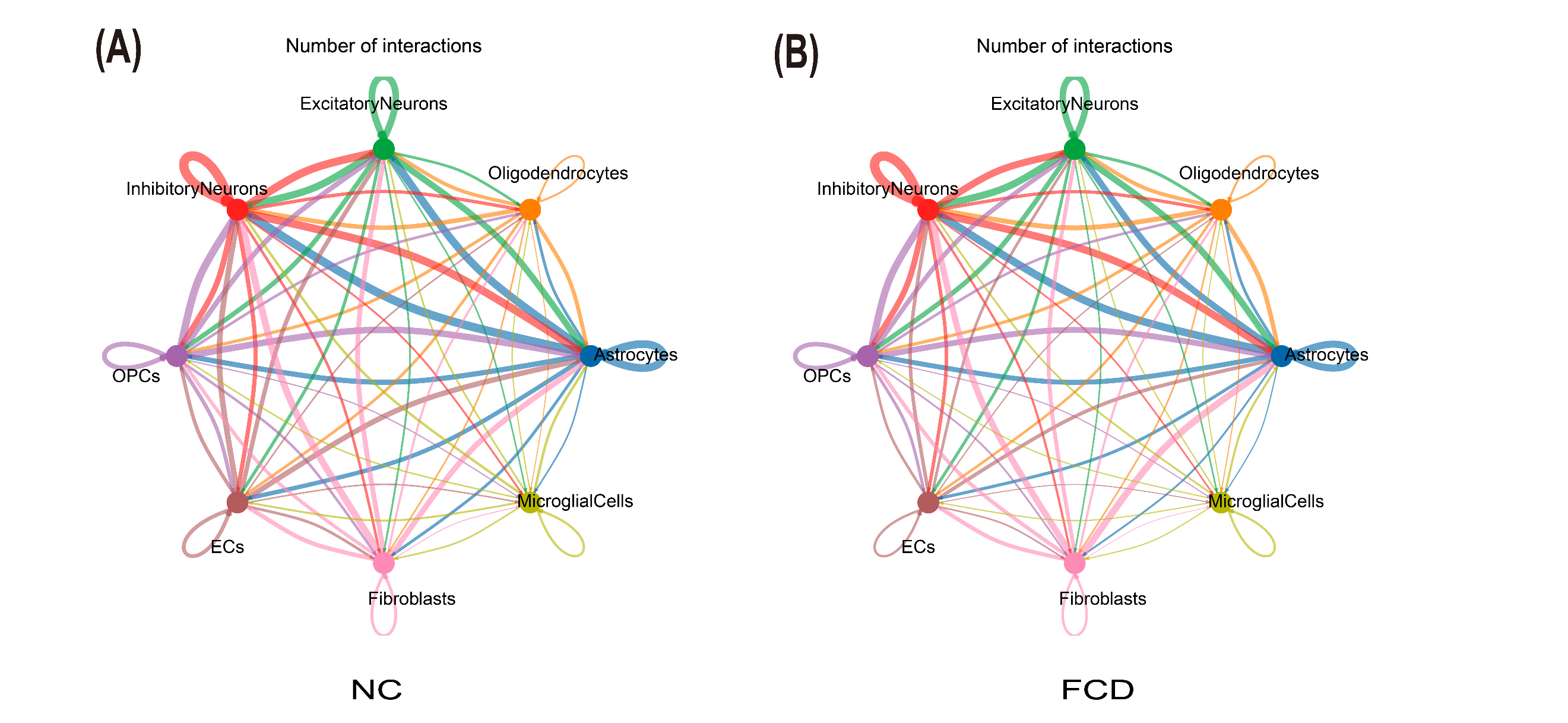


**SUPPLEMENTARY FIGURE 8. CellChat analysis depicted the intercellular communication networks between FCD and normal controls (NC).** A comparative analysis of inferred interaction numbers between FCD (A) and NC (B) groups was conducted to systematically evaluate cell-cell signaling in the FCD microenvironment. In the chord diagrams, the connecting lines were colored according to the ligand-expressing sender cell type, and their thickness was scaled proportionally to the number of interaction pairs.
